# CT analysis of thoracolumbar body composition for estimating whole-body composition

**DOI:** 10.1186/s13244-023-01402-z

**Published:** 2023-04-24

**Authors:** Jung Hee Hong, Hyunsook Hong, Ye Ra Choi, Dong Hyun Kim, Jin Young Kim, Jeong-Hwa Yoon, Soon Ho Yoon

**Affiliations:** 1 Department of Radiology, Dongsan Hospital, Keimyung University College of Medicine, Daegu, Korea; 2 Medical Research Collaborating Center, Seoul National University Hospital, Seoul, Korea; 3 Department of Radiology, Seoul Metropolitan Government-Seoul National University Boramae Medical Center, Seoul, Korea; 4 Institute of Health Policy and Management, Medical Research Center, Seoul National University, Seoul, Korea; 5 Department of Radiology, Seoul National University College of Medicine, Seoul National University Hospital, 101 Daehak-ro, Chongno-gu, Seoul, 03080 Republic of Korea

**Keywords:** Body composition, Computed tomography, Muscle, Visceral fat, Subcutaneous fat

## Abstract

**Background:**

To evaluate the correlation between single- and multi-slice cross-sectional thoracolumbar and whole-body compositions.

**Methods:**

We retrospectively included patients who underwent whole-body PET–CT scans from January 2016 to December 2019 at multiple institutions. A priori-developed, deep learning-based commercially available 3D U-Net segmentation provided whole-body 3D reference volumes and 2D areas of muscle, visceral fat, and subcutaneous fat at the upper, middle, and lower endplate of the individual T1–L5 vertebrae. In the derivation set, we analyzed the Pearson correlation coefficients of single-slice and multi-slice averaged 2D areas (waist and T12–L1) with the reference values. We then built prediction models using the top three correlated levels and tested the models in the validation set.

**Results:**

The derivation and validation datasets included 203 (mean age 58.2 years; 101 men) and 239 patients (mean age 57.8 years; 80 men). The coefficients were distributed bimodally, with the first peak at T4 (coefficient, 0.78) and the second peak at L2-3 (coefficient 0.90). The top three correlations in the abdominal scan range were found for multi-slice waist averaging (0.92) and single-slice L3 and L2 (0.90, each), while those in the chest scan range were multi-slice T12–L1 averaging (0.89), single-slice L1 (0.89), and T12 (0.86). The model performance at the top three levels for estimating whole-body composition was similar in the derivation and validation datasets.

**Conclusions:**

Single-slice L2–3 (abdominal CT range) and L1 (chest CT range) analysis best correlated with whole-body composition around 0.90 (coefficient). Multi-slice waist averaging provided a slightly higher correlation of 0.92.

**Supplementary Information:**

The online version contains supplementary material available at 10.1186/s13244-023-01402-z.

## Key points

- In single-slice analysis, the L2–3 and L1 levels had the closest correlations with whole-body composition.
- Multi-slice waist averaging (0.92; correlation) showed a better correlation than the L2–3 single-slice analysis (0.90) in the abdomen.
- Multi-slice T12–L1 averaging (0.89) provided a comparable correlation to the L1 level in the chest (0.89).

**Supplementary Information:**

The online version contains supplementary material available at 10.1186/s13244-023-01402-z.

## Introduction

Body composition, defined as the proportion of fat and muscle in the body, is an important modifiable risk factor associated with the clinical outcomes of chronic [1–4] and malignant diseases [5–7], as well as obesity-related health risks [8–10]. Several methods are used to assess body composition, including dual X-ray absorptiometry and bioelectrical impedance [11–13]. Nevertheless, cross-sectional CT and MR imaging are considered the gold standards because tissue can be directly separated on cross-sectional CT and MR images [14–20].

CT and MRI-based body composition analysis has been typically conducted using single slices because segmentation is laborious and time-consuming. Early studies analyzed the correlation between cross-sectional body composition in sparse lumbar levels and whole-body composition and showed the best correlation for the L3 vertebral level [21, 22]. Subsequently, many studies conducting CT-based body composition analysis at the L3 level identified numerous clinical implications of CT body composition analysis [21–27]. Furthermore, deep neural networks were recently developed for automatic single-slice segmentation of L3 body composition, enabling large-data analysis. However, chest CT scans do not include the L3 vertebra, and several vertebral levels, such as T4, T8, T12, and L1, have been suggested for body composition analysis instead of L3 [28, 29].

Body composition can vary in the craniocaudal direction, and the following questions have remained underexplored: a) how well body composition at individual thoracolumbar vertebral levels represents whole-body composition, particularly for chest CT scans; b) whether cross-sectional body composition may differ within a vertebral level; and c) whether multi-slice cross-sectional CT analysis is better correlated with whole-body composition than single-slice CT analysis.

This study aimed to evaluate the correlation between cross-sectional thoracolumbar body composition and whole-body composition.

## Materials and methods

The institutional review board of the participating hospitals approved this retrospective study and waived the requirement for patients’ informed consent.

### Study population

**Fig. 1:**
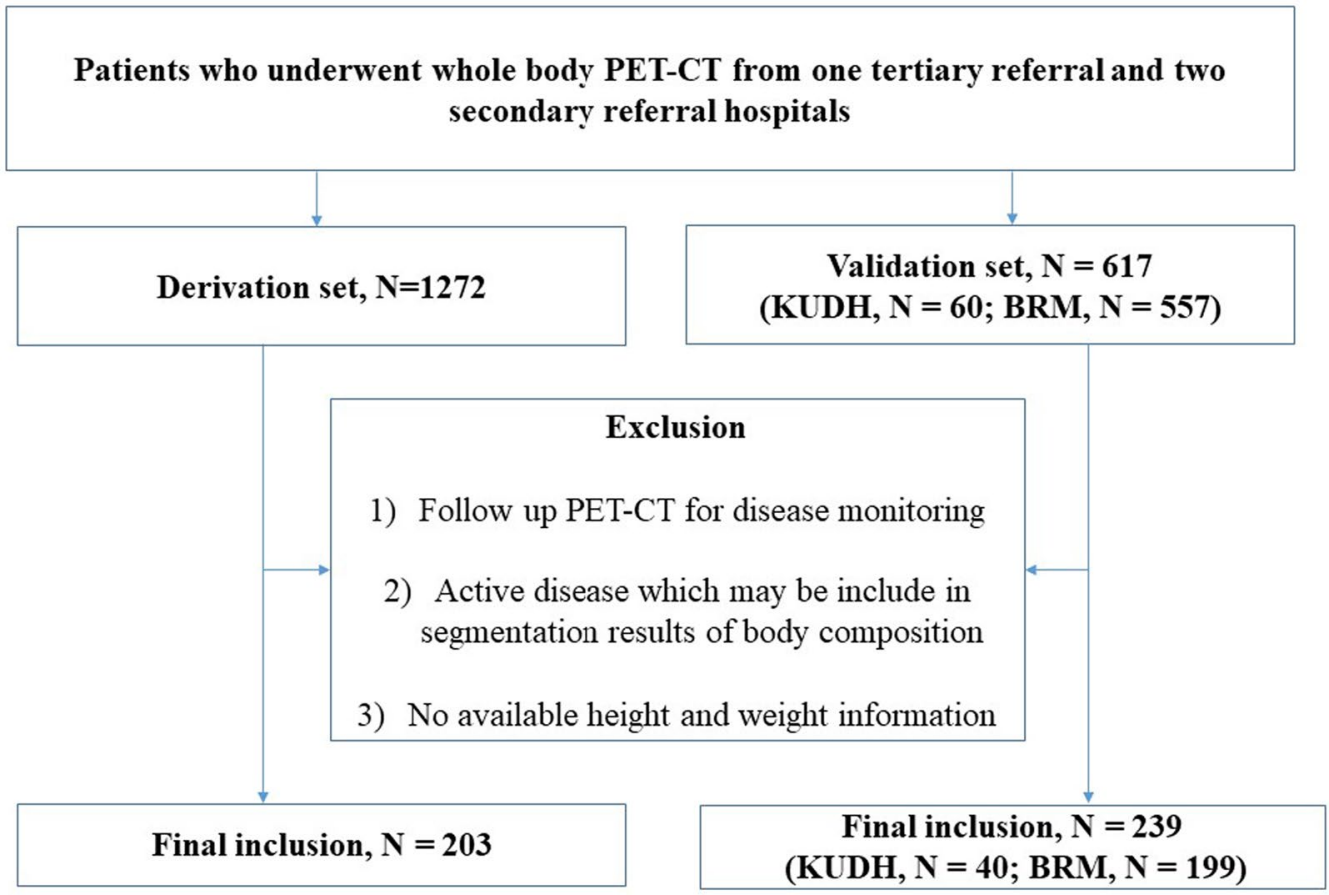
Patient inclusion flowchart

The study populations were collected retrospectively from one tertiary referral hospital (SNUH; derivation set) between January 2016 and December 2019 and two secondary referral hospitals (BRM and KUDH; validation set) with the same eligibility criteria (Fig. 1). The inclusion criteria were: (a) adult patients, (b) undergoing a baseline PET–CT scan covering the entire body from hand tip to toe, and (c) available height and weight information. The exclusion criteria were: (a) follow-up PET–CT for disease monitoring, (b) active disease that could be included in the segmentation results of body composition, and (c) no available height and weight information.

A study coordinator collected patients’ age, sex, weight, and height information. Body mass index (BMI) was calculated as the weight in kilograms divided by the square of the height in meters (kg/m^2^).

### PET/CT acquisition

All patients underwent PET–CT using integrated PET–CT scanners (Biograph Truepoint or mCT40, Siemens Healthineers; Gemini TF scanner, Philips Healthcare; Discovery STE, General Electric Healthcare). Patients fasted for at least 6 h, and FDG (5.18 MBq/kg) was administered intravenously. Images were acquired approximately 60 min after injection. Patients were examined in the supine position with the arm down. A CT scan (40 mA and 120 kVp) was performed for attenuation correction without contrast enhancement, and PET images were acquired from the skull base to the toe. The CT images were reconstructed using a 512 × 512 matrix in combination with a 50-cm field of view and a 3-mm slice thickness.

### Body composition segmentation

**Fig. 2:**
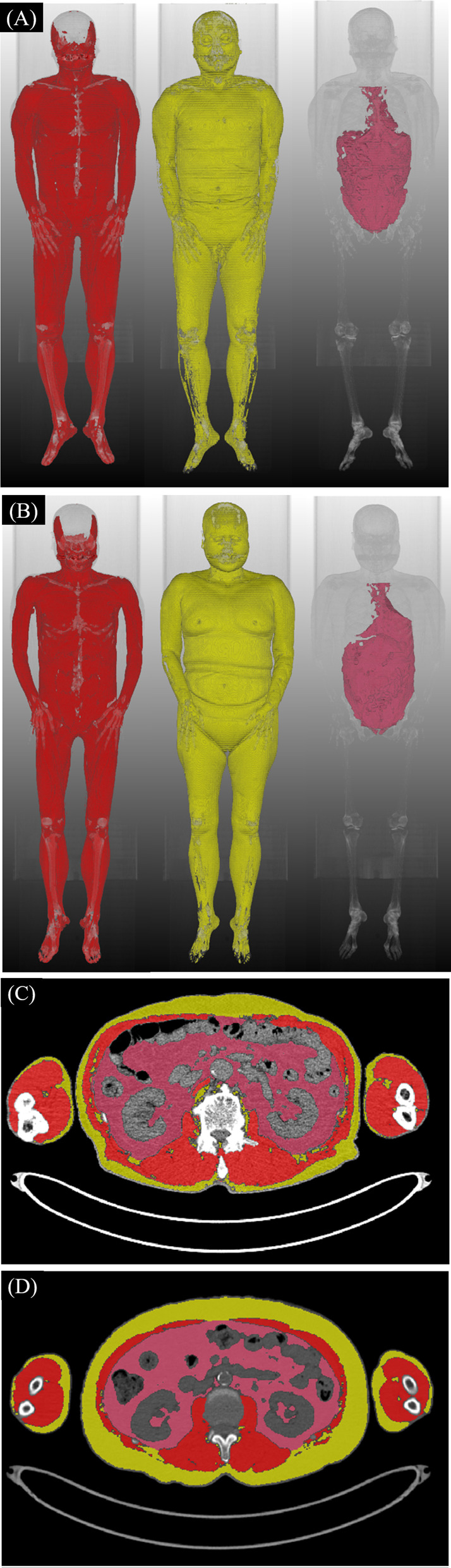
Representative whole-body 3D and cross-sectional CT images of segmented body composition in a 77-year-old male (**A**, **C**) and a 68-year-old female (**B**, **D**) patients. Whole-body 3D (**A**, **B**) and cross-sectional images at the L3 level (**C**, **D**) show differences in the distribution of muscle (purple), subcutaneous fat (yellow), and intraabdominal fat (red) by sex. Thoracolumbar cross-sectional areas of muscle, subcutaneous, and visceral fat were assessed from the trunk after excluding those tissues in the arms

CT images were processed using commercially available software (DeepCatch, version 1.x.x.x; MEDICAL IP Co., Ltd., Seoul, Korea) for the automatic volumetric segmentation of whole-body CT images. The software contained 2D and 3D U-Net that segmented CT images into seven classes: skin, muscle, bone, abdominal visceral fat (VF), subcutaneous fat (SF), internal organs/vessels, and central nervous system. A total of 39,268 image slices were used to develop the networks, and 3D U-Net provided an average dice score of 96.8–99.2% for muscle, 95.1–98.9% for VF, and 97.1–99.7% for SF (Fig. 2) in the domestic validation sets, respectively [30]. The chest radiologist (J.H.H., with 7 years of experience in CT interpretation) visually inspected the segmentation results to ensure the accuracy of the reference standard by the same software and confirmed that no further adjustments were required in all cases.

In the 3D U-Net’s segmentation, the intrathoracic VF was included in the SF mask. Accordingly, a chest radiologist (J.H.H., with 7 years of experience in CT interpretation) separated the intrathoracic VF from the SF mask in all cases and revised the U-Net’s segmentation results of muscle, abdominal fat, VF, and SF if necessary using same software. Intrathoracic VF was defined as the fat enclosed by the inner aspect of the sternum, lung, and spines, extending from the thoracic apex to the diaphragm [31]. The segmented intrathoracic VF was merged into the abdominal VF to represent the total VF [32].

The reference whole-body composition comprised whole-body volumes of muscle, VF, and SF. Single-slice segmented 2D areas of muscles, VF, and SF in the trunk were calculated at the upper endplate, lower endplate, and mid-vertebra between T1-L5. If the vertebral body was tilted, it was measured based on the slice containing the center of the anteroposterior plane (Additional file 1: Fig. S1).

We assumed that the chest CT scans covered the area from the upper endplate of T1 to the lower endplate of L1, while abdominal CT scans extended from the upper endplate of T10 to the lower endplate of L5 [33]. Multi-slice averaging was performed at T12–L1 (from the T12 upper endplate to the L1 lower endplate) for the chest CT range and the waist (from the lowest rib to the iliac crest) for the abdominal CT range.

### Statistical analysis

The Pearson’s correlation coefficient was used to assess the linear associations between reference (3D whole-body composition volume) and single-slice or multi-slice averaged 2D areas, and the 95% confidence interval was derived based on 1000 bootstrap resamples. We analyzed the coefficients of muscle, VF, and SF separately in each sex. In addition, we estimated overall correlation coefficients in the three compositions to identify the top three levels representing whole-body muscle, VF, and SF simultaneously. The coefficients were estimated with a linear mixed model, which accounts for dependency among observations within each individual, as proposed by Hamlett et al. [34]. In the analysis, measurements standardized by sex were used to make scales of measurements from the three compositions comparable, and the standardization was done as follows:

$$Z - {\text{score}} = \frac{{\left( {{\text{individual}}\;{\text{observation}}{-}{\text{average}}\;{\text{in}}\;{\text{the}}\;{\text{corresponding}}\;{\text{sex}}} \right)}}{{{\text{standard}}\;{\text{deviation}}\;{\text{in}}\;{\text{the}}\;{\text{corresponding}}\;{\text{sex}}}}$$

To build prediction models for whole-body composition in the derivation dataset, linear regression was used with the natural logarithm of whole-body composition as the outcome, and all predicted values were back-transformed to the original scale. The candidate predictors were the natural logarithms of body composition on CT slices and participants’ sex, age, height, and weight (Additional file 1: Text). Prediction models were determined by applying backward elimination based on the Akaike information criterion. Prediction models’ performance was assessed in terms of *R*^2^, the root-mean-squared error (RMSE), mean absolute error (MAE), calibration slope, and calibration-in-the-large. To assess the performance of prediction models in the derivation dataset, optimism-corrected statistics were estimated based on 1000 bootstrap samples. The prediction models were externally validated using data from BRM and KUDH. In the external validation, the models were updated by re-estimating intercepts to account for differences in whole-body composition distribution between the derivation and validation datasets (Additional file 1: Text).

A *P*-value less than 0.05 was considered to indicate statistical significance. All statistical analyses were performed using R software (version 4.1http://www.R-project.org/).

## Results

### Baseline characteristics

**Fig. 3:**
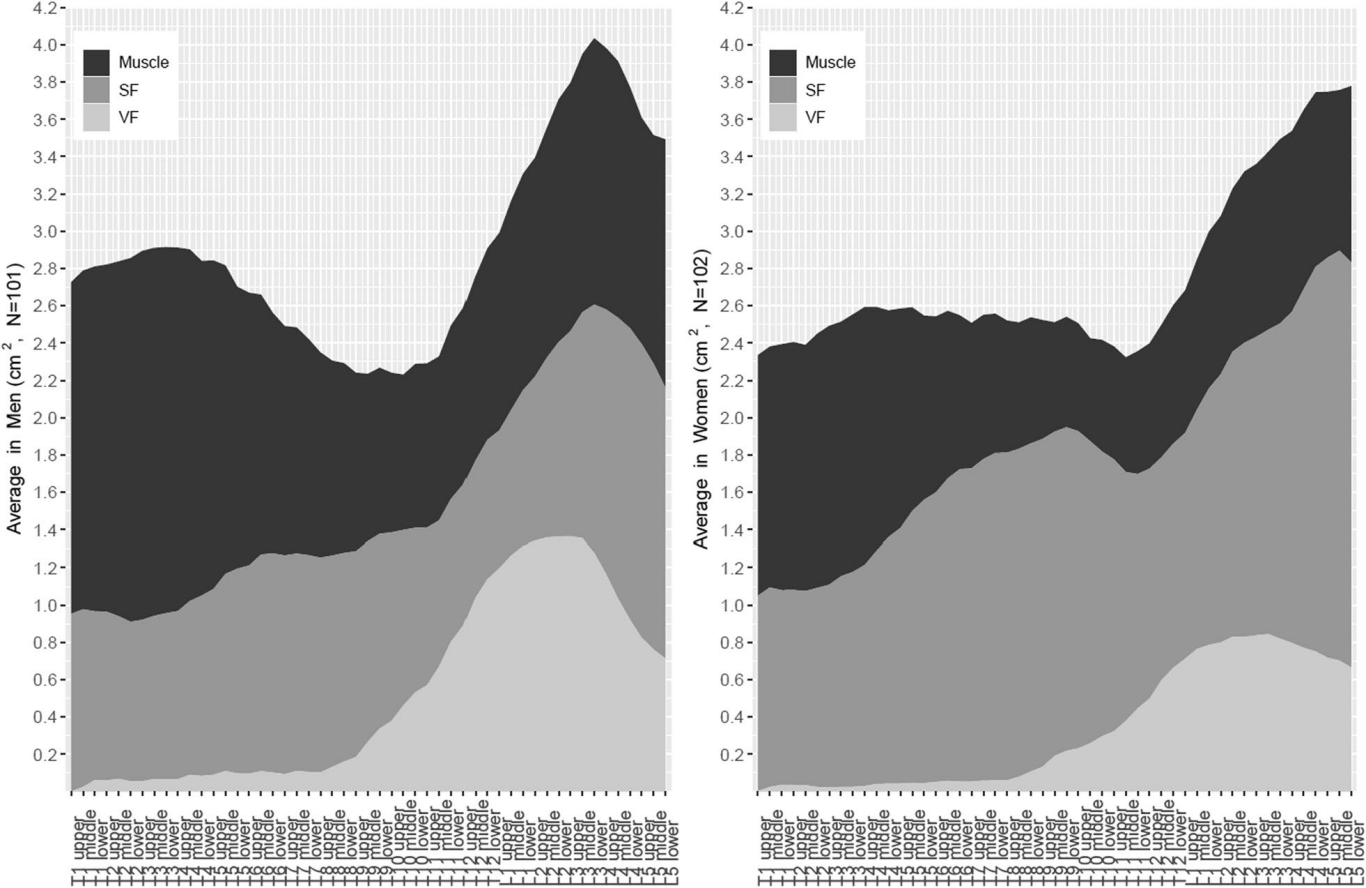
Averaged cross-sectional areas of muscle, subcutaneous fat (SF), and visceral fat (VF) at thoracolumbar levels in both sexes

Of the 1272 patients and 617 patients in derivation and validation dataset, the derivation dataset included 203 patients (mean age 58.2 years; 101 men; mean BMI 23.9 kg/m^2^) and the validation dataset included 239 patients from two external hospitals (Fig. 1 and Table 1): 199 patients (mean age 56.5 years; 61 men; mean BMI 23.4 kg/m^2^) and 40 patients (mean age 64.2 years; 19 men; mean BMI 24.0 kg/m^2^), respectively. The thoracolumbar body composition was differently distributed by sex (Fig. 3 and Additional file 1: Table S1): The summed amount of muscle, VF, and SF was highest at L3 in men and L5 in women. The muscle amount was largest at T1–3 in both sexes (male 1.77–1.97 cm^2^; female 1.29–1.38 cm^2^). The second-largest amount existed at L3 in men (1.43 cm^2^), while the amount was relatively consistent across the other T–L levels in women. The amount of SF was much larger in women than in men and peaked in L5 in both sexes (male, 1.57 cm^2^; female, 2.19 cm^2^). VF remained negligible at T1–T8 and then gradually increased and plateaued at L1–L3 (peak value, male, 1.36 cm^2^; female, 0.84 cm^2^).

**Table 1:** Participants’ characteristics in the derivation and validation datasets

	Derivation dataset			External validation dataset				
	Male (*n* = 101)	Female (*n* = 102)	All (*n* = 203)	Male (*n* = 80)	Female (*n* = 159)	All (*n* = 239)	BRM (*n* = 199)	KUDH (*n* = 40)
Age	59.3 ± 14.8	57.1 ± 16.3	58.2 ± 15.5	59.9 ± 13.8	56.7 ± 12.5	57.8 ± 13.0	56.5 ± 12.5	64.2 ± 13.8
Height (cm)^a^	169.6 ± 6.9	157.4 ± 6.5	163.5 ± 9.1	166.4 ± 7.2	156.8 ± 6.7	160.0 ± 8.2	159.9 ± 8.3	160.3 ± 8.1
Weight (kg)^a^	69.9 ± 10.8	58.3 ± 9.2	64.1 ± 11.6	65.4 ± 11.4	57.7 ± 8.8	60.4 ± 10.4	59.9 ± 10.1	61.9 ± 11.47
Body mass index^a^	24.3 ± 3.3	23.5 ± 3.5	23.9 ± 3.4	23.5 ± 3.2	23.5 ± 3.3	23.5 ± 3.3	23.4 ± 3.3	24.0 ± 3.2
< 18.5	2 (2.0%)	9 (8.8%)	11 (5.4%)	5 (6.3%)	9 (5.7%)	14 (5.9%)	13 (6.5%)	1 (2.5%)
18.5–22.9	38 (37.6%)	37 (36.3%)	75 (36.9%)	29 (36.3%)	65 (40.9%)	94 (39.3%)	79 (39.7%)	15 (37.5%)
23–24.9	25 (24.8%)	23 (22.5%)	48 (23.6%)	20 (25.0%)	31 (19.5%)	51 (21.3%)	44 (22.1%)	7 (17.5%)
25–29.9	30 (29.7%)	30 (29.4%)	60 (29.6%)	25 (31.3%)	52 (32.7%)	77 (32.2%)	61 (30.7%)	16 (40.0%)
≥ 30	6 (5.9%)	3 (2.9%)	9 (4.4%)	1 (1.3%)	2 (1.3%)	3 (1.3%)	2 (1.0%)	1 (2.5%)
*Body composition (liter)*^*a*^								
Whole-body muscle	23.9 ± 4.5	16.4 ± 2.6	20.1 ± 5.2	19.7 ± 4.0	14.7 ± 2.6	16.4 ± 3.9	16.3 ± 3.5	17.1 ± 5.4
Whole-body visceral Fat	3.6 ± 1.9	2.3 ± 1.4	3.0 ± 1.8	3.0 ± 1.7	2.1 ± 1.2	2.4 ± 1.4	2.3 ± 1.4	3.0 ± 1.4
Whole-body subcutaneous fat	13.7 ± 5.2	18.4 ± 5.4	16.1 ± 5.8	9.7 ± 4.8	14.3 ± 4.5	12.8 ± 5.1	12.6 ± 5.1	13.3 ± 5.1

^a^Mean values ± standard deviation are presented

### T1–L5 correlation coefficients for muscle, VF, and SF

**Fig. 4:**
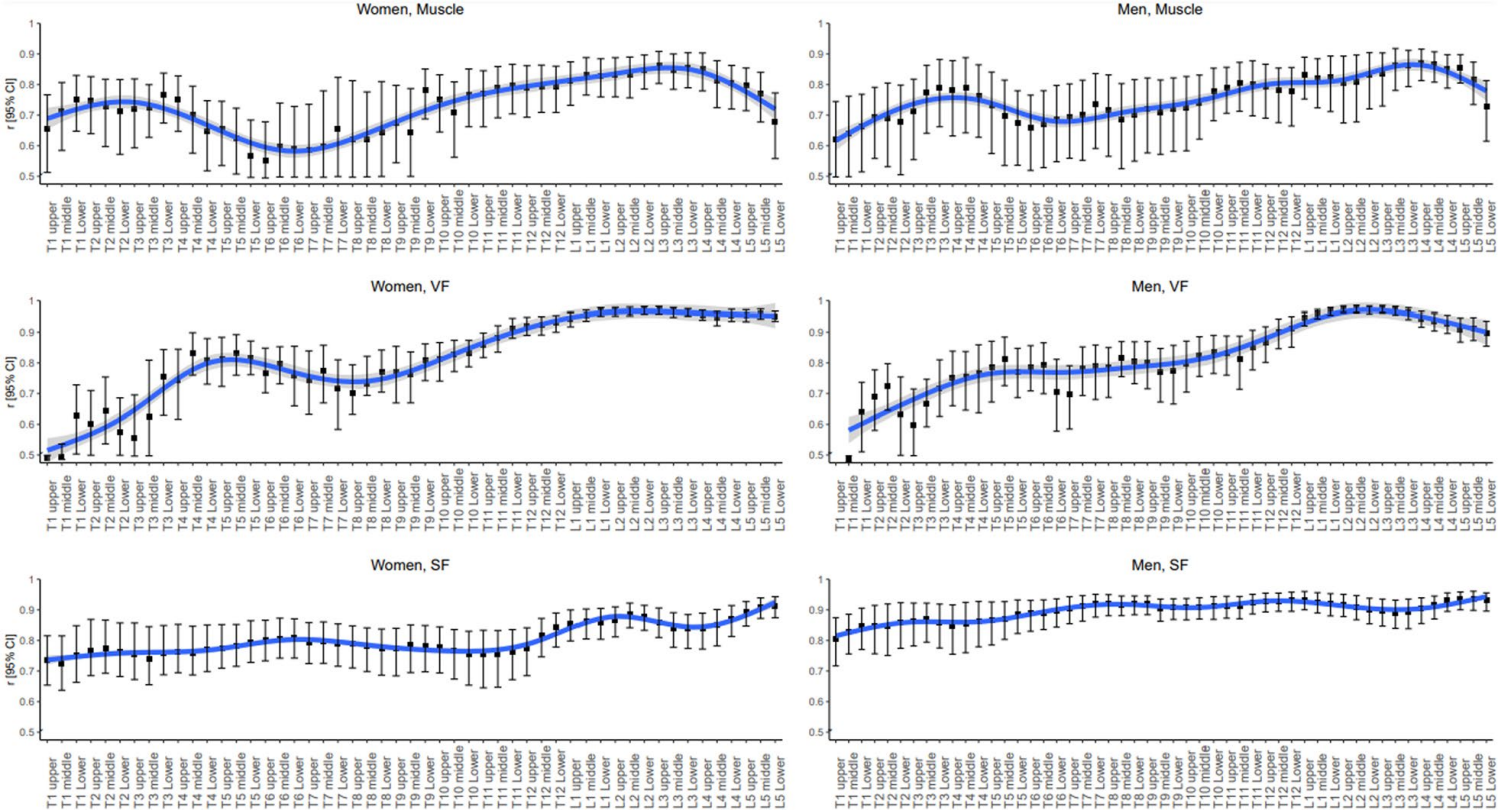
Bar graphs of Pearson correlation coefficients for whole-body composition and 95% confidence intervals based on 1000 bootstrap resamples

The Pearson correlation coefficients of muscle, VF, and SF using 1000 bootstrap resamples are summarized in Fig. 4 and Additional file 1: Table S2.

The craniocaudal trend (T1–L5) in the coefficients for muscle presented a bimodal shape regardless of sex: the first peak was around the T3–T4 level (coefficients 0.79 in men and 0.76 in women) followed by a decrease and nadir at T6 (coefficients, 0.66 in men and 0.55 in women). The coefficients then gradually increased and eventually peaked at L3–L4 (coefficients, 0.87 in men and 0.86 in women).

The T1–L5 craniocaudal trend of the VF coefficients in women also showed a bimodal shape: the first peak was around T4–5 (coefficient, 0.83), followed by a decrease and nadir at T8 (coefficient, 0.70). Then, the coefficient was over 0.90 at T12, followed by a peak at L2–3 (coefficient, 0.97). In men, the coefficients resembled a parabolic curve with a skewed peak at the lumbar levels. The coefficients increased, with some degree of fluctuation, but remained 0.80 or smaller at T1–T9 and increased to over 0.90 at T12 with a peak at L2–3 (0.98).

The SF coefficients in women at T1–L5 tended to be lower than in men by 0.05–0.1 in general. The coefficients plateaued at 0.8 or less at T1–T11. Then, they reached around 0.85 at most lumbar levels, with a peak at L5 (coefficient, 0.91). The coefficients in men were 0.80 at T1 and reached around 0.90 at T6, with mild fluctuations across vertebrae and peaks at T12 and L5 (0.93).

### Best single-slice and multi-slice averaging in the chest and abdominal CT ranges

**Fig. 5:**
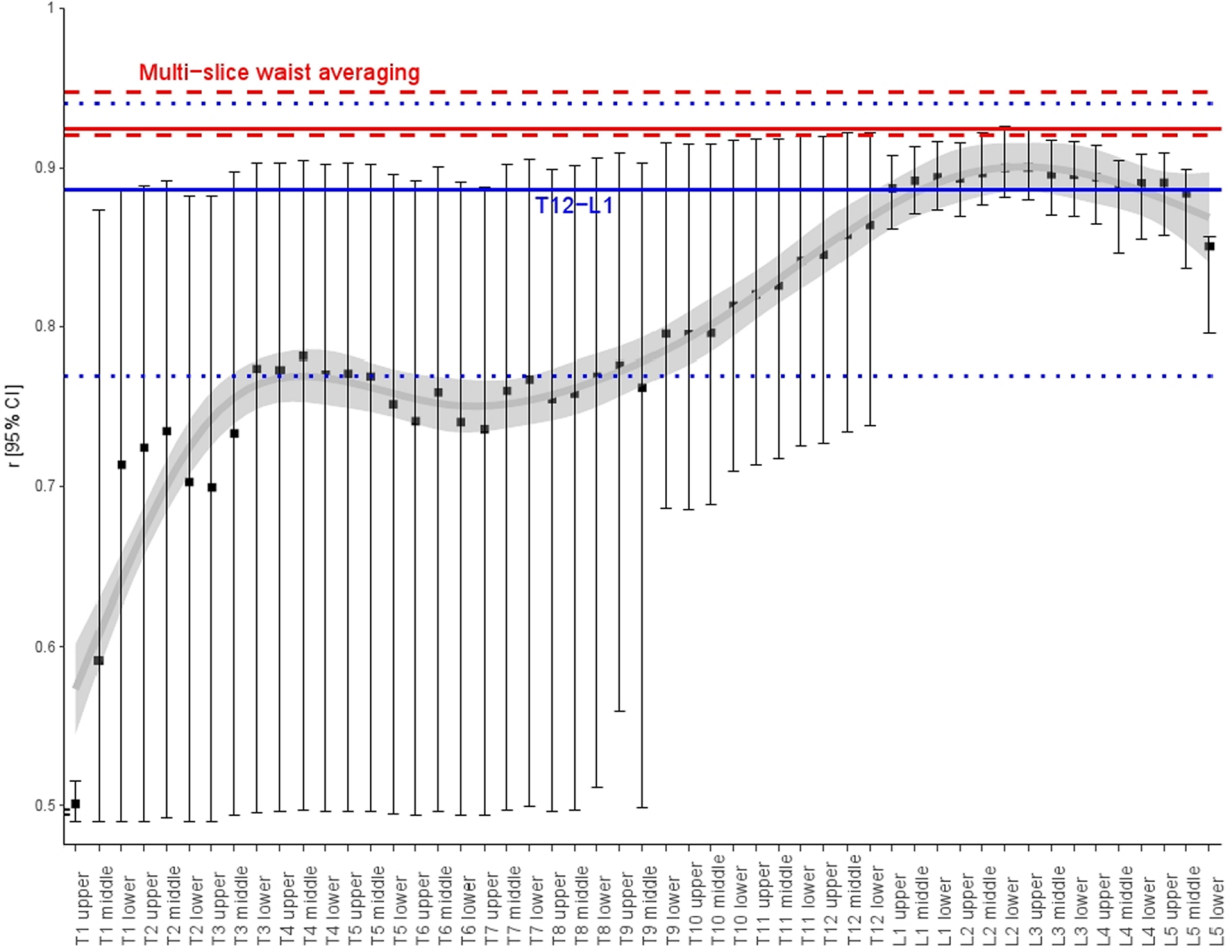
Bar graph plotting correlation coefficients for whole-body composition after standardizing muscle, visceral fat, and subcutaneous fat measurements by sex and confidence intervals based on 1000 bootstrap resamples

The correlation coefficients in each composition are summarized in Fig. 4 and Additional file 1: Table S2, and coefficient in three compositions is presented in Fig. 5 and Additional file 1: Table S3.

The T1–L5 craniocaudal trend of the overall coefficients had a bimodal shape: the coefficient began around 0.6 at T1, followed by the first peak at T4 (coefficient, 0.78). The coefficient mildly decreased at T6–7 to 0.74 and then gradually increased toward the second peak at L2–3 (coefficient, 0.90).

The top three correlated levels in the abdominal scan range were multi-slice waist averaging (0.92), followed by single-slice L3 and L2 (0.90, each), and those in the chest scan range were multi-slice T12–L1 averaging (0.89) and single-slice L1 (0.89), followed by T12 (0.86). The coefficients within vertebrae were similar regardless of whether they were obtained at the upper, middle, or lower endplate levels. Nevertheless, within the vertebrae, the best single-slice levels were regarded as the L3 upper endplate, L2 lower endplate, T12 lower endplate, and L1 lower endplate because these levels produced the highest 95% confidence intervals of coefficients.

### Prediction models in abdominal and thoracic CT

Prediction models to estimate whole-body muscle, VF, and SF were derived from the top three single-slice and multi-slice cross-sectional analyses. In the abdominal scan range, multi-slice waist averaging, the single-slice L3 upper endplate, and the single-slice L2 lower endplate were chosen. In the chest scan range, multi-slice T12–L1 averaging, the single-slice L1 upper endplate, and the single-slice T12 lower endplate were selected (Table 2 and Additional file 1: Tables S4–6). The L1 upper endplate was selected because a chest CT scan may not cover the entire L1 vertebra, and the correlation coefficients within the L1 vertebra did not significantly differ.

**Table 2:** Model performance for predicting whole-body composition in the internal dataset

	Multi-slice averaging in the waist	Single-slice L2 lower endplate	Single-slice L3 upper endplate	Multi-slice averaging in T12–L1	Single-slice T12 lower endplate	Single-slice L1 upper endplate
*Muscle*						
*R*^2^	0.94	0.92	0.92	0.91	0.90	0.92
RMSE (L)	1.34	1.51	1.53	1.57	1.67	1.46
MAE (L)	1.03	1.07	1.09	1.10	1.20	1.05
Calibration-in-the-large (L)	0.11 (− 0.61, 0.82)	0.09 (− 0.70, 0.89)	0.15 (− 0.66, 0.96)	− 0.02 (− 0.86, 0.83)	0.02 (− 0.89, 0.92)	− 0.10 (− 0.89, 0.69)
Calibration slope	1.00 (0.96, 1.03)	1.00 (0.96, 1.04)	0.99 (0.96, 1.03)	1.00 (0.96, 1.04)	1.00 (0.96, 1.05)	1.01 (0.97, 1.05)
*Visceral fat*						
*R*^2^	0.96	0.94	0.94	0.92	0.87	0.91
RMSE (L)	0.38	0.45	0.42	0.49	0.65	0.54
MAE (L)	0.26	0.32	0.30	0.34	0.44	0.38
Calibration-in-the-large (L)	− 0.01 (− 0.11, 0.08)	− 0.10 (− 0.21, 0.02)	− 0.08 (− 0.19, 0.03)	− 0.03 (− 0.16, 0.09)	0.06 (− 0.11, 0.24)	− 0.08 (− 0.22, 0.07)
Calibration slope	1.01 (0.98, 1.04)	1.05 (1.01, 1.09)	1.04 (1.01, 1.08)	1.03 (0.99, 1.06)	1.00 (0.95, 1.05)	1.05 (1.01, 1.09)
*Subcutaneous fat*						
*R*^2^	0.89	0.87	0.86	0.90	0.89	0.89
RMSE (L)	1.97	2.07	2.15	1.87	1.94	1.96
MAE (L)	1.45	1.54	1.60	1.42	1.47	1.49
Calibration-in-the-large (L)	0.86 (0.09, 1.62)	0.89 (0.09, 1.70)	0.99 (0.15, 1.83)	0.51 (− 0.23, 1.25)	0.55 (− 0.22, 1.32)	0.54 (− 0.24, 1.32)
Calibration slope	0.95 (0.91, 1.00)	0.95 (0.90, 1.00)	0.94 (0.90, 0.99)	0.97 (0.93, 1.02)	0.97 (0.93, 1.02)	0.97 (0.93, 1.02)

*RMSE *Root-mean-squared error, *MAE* Mean absolute error

Data in parentheses indicate 95% confidence intervals

The prediction model derived from multi-slice waist averaging provided the best estimation for muscle, VF, and SF in the internal dataset (*R*^2^, 0.89–0.96; RMSE, 0.38–1.97 L; MAE, 0.26–1.45 L; calibration-in-the-large, of − 0.01–0.86 L; calibration slope, 0.95–1.01) (Table 2 and Additional file 1: Fig. S2). For the abdominal scan range, the single-slice L2 lower endplate (*R*^2^, 0.87–0.94; calibration slope, 0.95–1.05) and L3 upper endplate (*R*^2^, 0.86–0.94; 0.94–1.04) were slightly worse than multi-slice waist averaging in all performance measures, including RMSE and MAE. For the chest scan range, the model performance was similar between multi-slice T12–L1 averaging (*R*^2^, 0.90–0.92; calibration slope, 0.97–1.03) and the single-slice L1 upper endplate (*R*^2^, 0.89–0.92; calibration slope, 0.97–1.05). The single-slice T12 lower endplate provided slightly lower performance (*R*^2^, 0.87–0.90; calibration slope, 0.97–1.00) and larger RMSE and MAE in general.

In the external validation, the performances of updated models were improved than original models, and a similar order of model performance was replicated in the external dataset. Multi-slice waist averaging provided the best estimations for muscle, VF, and SF (*R*^2^, 0.90–0.94; RMSE, 0.34–1.63 L; MAE, 0.25–1.23 L; calibration-in-the-large, − 0.02 to − 0.30 L; calibration slope, 1.01–1.05) (Table 3 and Additional file 1: Fig. S3). For the abdominal scan range, the single-slice L2 lower endplate (*R*^2^, 0.88–0.92; calibration slope, 0.84–1.10) and L3 upper endplate (*R*^2^, 0.88–0.92; calibration slope 1.00–1.11) provided slightly lower performance. For the chest scan range, similar performance was found between multi-slice T12–L1 averaging (*R*^2^, 0.89–0.92; calibration slope, 0.88–1.01) and the single-slice L1 upper endplate (*R*^2^, 0.88–0.90; calibration slope, 0.96–1.02). The single-slice T12 lower endplate provided a slightly lower *R*^2^ (0.87–0.90) for muscle, VF, and SF and resulted in a slightly larger RMSE and MAE for muscle and VF.

**Table 3:** Model performance for predicting whole-body composition in the external dataset

	Multi-slice averaging in the waist	Single-slice L2 lower endplate	Single-slice L3 upper endplate	Multi-slice averaging in T12–L1	Single-slice T12 lower endplate	Single-slice L1 upper endplate
*Before updating models*						
Muscle						
*R*^2^	0.48	0.41	0.45	0.42	0.46	0.39
RMSE (L)	2.79	2.98	2.89	2.96	2.86	3.04
MAE (L)	2.51	2.64	2.58	2.68	2.54	2.71
Calibration-in-the-large (L)	− 0.04 (− 0.71, 0.63)	0.38 (− 0.38, 1.15)	0.13 (− 0.63, 0.88)	− 0.25 (− 1.01, 0.51)	− 0.05 (− 0.87, 0.77)	0.12 (− 0.67, 0.92)
Calibration slope	0.87 (0.84, 0.90)	0.84 (0.80, 0.88)	0.86 (0.82, 0.90)	0.88 (0.84, 0.91)	0.87 (0.83, 0.91)	0.85 (0.81, 0.90)
Visceral fat						
*R*^2^	0.94	0.93	0.93	0.9	0.87	0.89
RMSE (L)	0.35	0.38	0.38	0.45	0.51	0.47
MAE (L)	0.26	0.3	0.3	0.33	0.37	0.36
Calibration-in-the-large (L)	− 0.02 (− 0.11, 0.07)	− 0.16 (− 0.26, − 0.06)	− 0.17 (− 0.27, − 0.06)	− 0.02 (− 0.12, 0.09)	0.01 (− 0.11, 0.13)	− 0.07 (− 0.18, 0.05)
Calibration slope	0.99 (0.95, 1.02)	1.03 (1.00, 1.07)	1.04 (1.01, 1.08)	0.93 (0.90, 0.97)	0.93 (0.89, 0.97)	0.96 (0.92, 1.00)
Subcutaneous fat						
*R*^2^	0.61	0.44	0.44	0.52	0.53	0.47
RMSE (L)	3.16	3.8	3.8	3.51	3.48	3.71
MAE (L)	2.7	3.4	3.4	3.02	2.96	3.2
Calibration-in-the-large (L)	− 0.30 (− 0.89, 0.29)	− 0.71 (− 1.31, − 0.10)	− 0.78 (− 1.40, − 0.16)	0.16 (− 0.39, 0.72)	0.35 (− 0.21, 0.91)	0.06 (− 0.53, 0.66)
Calibration slope	0.85 (0.81, 0.89)	0.84 (0.80, 0.87)	0.84 (0.81, 0.88)	0.80 (0.77, 0.84)	0.80 (0.76, 0.83)	0.80 (0.77, 0.84)
*After updating models*						
Muscle						
*R*^2^	0.91	0.88	0.89	0.89	0.87	0.88
RMSE (L)	1.15	1.33	1.29	1.28	1.38	1.35
MAE (L)	0.81	0.88	0.86	0.88	0.93	0.93
Calibration-in-the-large (L)	− 0.04	0.38	0.13	− 0.25	− 0.05	0.12
	(− 0.71, 0.63)	(− 0.38, 1.15)	(− 0.63, 0.88)	(− 1.01, 0.51)	(− 0.87, 0.77)	(− 0.67, 0.92)
Calibration slope	1.01	0.84	1	0.88	1.01	1
	(0.97, 1.04)	(0.80, 0.88)	(0.95, 1.04)	(0.84, 0.91)	(0.96, 1.06)	(0.95, 1.04)
Visceral fat						
*R*^2^	0.94	0.92	0.92	0.92	0.88	0.9
RMSE (L)	0.34	0.4	0.41	0.39	0.48	0.45
MAE (L)	0.25	0.3	0.31	0.28	0.35	0.32
Calibration-in-the-large (L)	− 0.02	− 0.16	− 0.17	− 0.02	0.01	− 0.07
	(− 0.11, 0.07)	(− 0.26, − 0.06)	(− 0.27, − 0.06)	(− 0.12, 0.09)	(− 0.11, 0.13)	(− 0.18, 0.05)
Calibration slope	1.02	1.03	1.04	0.93	1.03	0.96
	(0.99, 1.05)	(1.00, 1.07)	(1.01, 1.08)	(0.90, 0.97)	(0.99, 1.08)	(0.92, 1.00)
Subcutaneous fat						
*R*^2^	0.9	0.89	0.88	0.9	0.9	0.89
RMSE (L)	1.63	1.71	1.76	1.57	1.6	1.67
MAE (L)	1.23	1.3	1.35	1.14	1.17	1.24
Calibration-in-the-large (L)	− 0.3	− 0.71	− 0.78	0.16	0.35	0.06
	(− 0.89, 0.29)	(− 1.31, − 0.10)	(− 1.40, − 0.16)	(− 0.39, 0.72)	(− 0.21, 0.91)	(− 0.53, 0.66)
Calibration slope	1.05	1.1	1.11	1.01	0.99	1.02
	(1.00, 1.09)	(1.05, 1.14)	(1.06, 1.15)	(0.97, 1.05)	(0.94, 1.03)	(0.97, 1.06)

*RMSE* Root-mean-squared error, *MAE* Mean absolute error

Data in parentheses indicate 95% confidence intervals

## Discussion

Using the up-to-date 3D U-Net for the volumetric segmentation of body composition, we assessed the distribution of muscle, SF, and VF in T1–L5 cross-sectional CT images and evaluated the correlations of those estimates with whole-body composition. In a single-slice analysis of abdominal and chest scans, the L2–3 and L1 levels had the closest correlations (0.90 and 0.89, respectively) with whole-body composition. Multi-slice waist averaging (0.92) showed a better correlation than the L2–3 single-slice analysis in the abdomen, and multi-slice T12–L1 averaging (0.89) provided a comparable correlation to the L1 level in the chest. The models built for estimating whole-body composition based on the top three levels estimated whole-body composition in a similar order for abdominal and chest scan ranges in the derivation and external validation sets.

Most CT studies have analyzed body composition using single slices at specific vertebral levels [21, 22, 35, 36]. The L3 vertebra are widely used, but it has rarely been specified where researchers made measurements within the vertebra. In the present study, the coefficients within the vertebrae of three body compartments (muscle, SF and SV) were similar regardless of the endplate levels in the top three levels, with the exception of occasional sharp differences (e.g., VF amount at T1, 0.25–0.64 in men; 0.26–0.63 in women). Therefore, assessing cross-sectional body composition at these vertebral levels would obviate the need to be concerned about which level within a vertebra should be measured.

L3 (and the lower L2 level) correlated best with the whole-body composition (correlation coefficients, 0.90), in accordance with prior studies [22, 35], but it is not routinely included in the chest CT scan range. In the chest scan range, our results showed the highest correlation coefficients of 0.89 for the single-slice L1 level and multi-slice T12–L1 averaging, whereas the upper to mid-thoracic spine levels represented by T4 and T8 yielded coefficients of around 0.77 with large confidence intervals. Considering the correlation coefficients of 0.90 in L2–3 and 0.89 in T12–L1, chest CT seems to have the potential to estimate whole-body composition comparably to abdominal CT [37].

Single-slice CT analysis has most often been used to estimate body composition rather than multi-slice cross-sectional CT analysis, which requires substantial effort to manually segment consecutive CT images. Nevertheless, earlier studies suggested that multi-slice CT analysis could estimate whole-body composition more accurately, considering variation in the body composition in the *Z*-axis and bowel shifting. In addition, selecting representative slices can cause intra- and inter-observer variability [16]. Recent advances in deep neural networks have enabled multi-slice body composition CT and our 3D U-Net able to process a single case less than a few minutes. Indeed, multi-slice waist averaging could alleviate the varying distribution [38], surpassing L3 in the abdominal scan range, and multi-slice T12–L1 averaging provided comparable correlations with L1 in the chest scan range. A low-dose chest CT scan typically limits the scan range confined to the lung parenchyma, and in our experience, one-fourth of low-dose chest CT scans did not cover L1 (unpublished). This results in significant missing data if a single-slice body composition analysis is performed at the L1 level in low-dose CT screening for lung cancer. However, multi-slice T12–L1 averaging can effectively complete body composition data acquisition, allowing for the identification of sarcopenic heavy smokers in a large-data low-dose CT scan analysis [39]. However, it should be acknowledged that the network’s results required an assistance of a radiologist for the confirmation and readjustment of the intrathoracic VF for this study.

Our study has several limitations. First, the number of subjects in the study population was relatively small and retrospectively collected at a limited number of institutions. Second, we excluded patients with abnormal lesions that would potentially interfere with the CT quantification of body composition. Almost all lesions excluded from the study were metastasis or primary cancer lesions. Those cases were inevitably excluded from the analysis since the margin of the lesions was less identifiable on non-contrast CT scans. Third, we did not analyze and compare body composition using other modalities, such as dual-energy X-ray absorptiometry or bioelectrical impedance analysis. Nevertheless, CT is regarded as the gold standard for body composition analysis, and radiologists confirmed the 3D U-Net’s segmentation results. Studies comparing with other modalities such as DEXA help further validate our results. Fourth, we used only non-contrast CT scans for body segmentation; this was inevitable because whole-body PET–CT scans are routinely performed without contrast in the participating institution, potentially causing segmentation errors in references. For instance, small vessels running distal to the thyroid, axilla, and proximal thighs could not be separated from the muscles masks, as radiologists or the network could not identify the small vessels on non-contrast CT scans. In addition, due to difference in PET/CT and CT protocols dedicated to the chest or abdomen, the spatial resolution of whole-body PET/CT scans is relatively low compared to CT scans. Despite this, using whole-body CT scans was deemed necessary in this study as CT or MR scan is considered the best modality for capturing body composition. Accordingly, our results might not apply to CT scans with different contrast or spatial resolution or MR scans and warrant further validation. Fifth, intramuscular fat was classified as subcutaneous fat. Sixth, the body fat distribution observed in our study may not be representative of the general population, as the retrospectively selected patients were relatively normal or slightly overweight patients. The number of subjects corresponding to obese and underweight is small, making it difficult to conduct a small group analysis in this data. We explored the relationship between whole-body composition and slice body composition through a scatter plot in the obese or underweight group compared to the normal group, but there was no significant deviation in all six areas (Additional file 1: Fig. S4). A larger-scale study that can adequately reflect the obesity levels of the entire population is needed. In conclusion, single-slice CT analysis best-estimated whole-body composition at the L2–3 level and L1 level for abdominal and chest CT ranges, respectively. Multi-slice averaging produced comparable to better results. These results confirm the utility of abdominal body composition CT analysis and show that chest CT analysis can potentially estimate whole-body composition reasonably, like an abdominal CT scan. Multi-slice averaging can alleviate a different distribution between CT slices.

## Supplementary Information

**Additional file 1:** Supplementary Text (statistical analysis), Supplementary Tables 1–6 and Supplementary Figures 1–4.

## Data Availability

The datasets used and/or analyzed during the current study are available from the corresponding author on reasonable request.

## Abbreviations

BMI: Body mass index
MAE: Mean absolute error
RMSE: Root-mean-squared error
SF: Subcutaneous fat
VF: Visceral fat
