# Supplementary material for: CT analysis of thoracolumbar body composition for estimating whole-body composition
Source: Insights Imaging. 2023 Apr 24;14:69. doi: 10.1186/s13244-023-01402-z (PMC10126176; doi:10.1186/s13244-023-01402-z)
Supplement: Supplementary file 1 — Additional file 1: Supplementary Text (statistical analysis), Supplementary Tables 1–6 and Supplementary Figures 1–4. [file 13244_2023_1402_MOESM1_ESM.pdf]

ELECTRONIC SUPPLEMENTARY MATERIAL

CT Analysis of Thoracolumbar Body Composition for Estimating Whole-body Composition

Supplementary text

Statistical analysis

When building the prediction models, to account for different relationships between whole-body composition and candidate predictors according to sex, interactions between sex and all the remaining candidate predictors were also considered as potential predictors. R^2 represents the proportion of variation in whole-body composition explained by the predictors, while both RMSE and MAE provide information on the average difference between observed and predicted values. Calibration slope and calibration-in-the-large reflect the degree of agreement between observed and predicted values, and good calibration is achieved when the calibration-in-the-large is close to 0 and the calibration slope is close to 1. In the external validation step, the models were updated by re-estimating the intercepts to account for differences in the whole-body composition distribution between the derivation and validation datasets. The performance of models before and after updating was evaluated as well.

Supplementary Table 1. Summary of averaged cross-sectional areas of muscle, subcutaneous fat (SF), and visceral fat (VF) at thoracolumbar levels in both sexes.

	Muscle slice mean (cm^2)		Subcutaneous fat slice mean (cm^2)		Visceral fat slice mean (cm^2)	
	**Female**	**Male**	**Female**	**Male**	**Female**	**Male**
**T1 upper***	1.29	1.77	1.05	0.95	0.00	0.00
**T1 middle***	1.29	1.81	1.07	0.95	0.02	0.02
**T1 lower***	1.31	1.84	1.04	0.90	0.04	0.06
**T2 upper***	1.32	1.86	1.05	0.90	0.04	0.06
**T2 middle***	1.32	1.90	1.04	0.87	0.03	0.07
**T2 lower***	1.36	1.94	1.07	0.86	0.02	0.06
**T3 upper***	1.38	1.97	1.09	0.87	0.02	0.06
**T3 middle***	1.36	1.97	1.13	0.87	0.02	0.07
**T3 lower***	1.38	1.96	1.15	0.89	0.03	0.07
**T4 upper***	1.38	1.94	1.19	0.90	0.03	0.07
**T4 middle***	1.30	1.88	1.25	0.93	0.04	0.09
**T4 lower***	1.22	1.79	1.32	0.97	0.04	0.08
**T5 upper***	1.18	1.76	1.36	1.00	0.04	0.09
**T5 middle***	1.09	1.65	1.45	1.06	0.05	0.11
**T5 lower***	0.99	1.51	1.51	1.10	0.05	0.10
**T6 upper***	0.94	1.46	1.55	1.12	0.05	0.10
**T6 middle***	0.90	1.39	1.62	1.16	0.06	0.11
**T6 lower***	0.83	1.28	1.67	1.18	0.05	0.10
**T7 upper***	0.78	1.22	1.67	1.17	0.05	0.09
**T7 middle***	0.77	1.21	1.72	1.17	0.06	0.11
**T7 lower***	0.75	1.15	1.75	1.16	0.06	0.11
**T8 upper***	0.71	1.10	1.75	1.15	0.06	0.10
**T8 middle***	0.68	1.04	1.75	1.13	0.08	0.13
**T8 lower***	0.68	1.01	1.75	1.12	0.11	0.16
**T9 upper***	0.64	0.95	1.75	1.10	0.13	0.19

**T9 middle***	0.59	0.90	1.73	1.07	0.19	0.27
**T9 lower***	0.59	0.89	1.73	1.04	0.22	0.34
**T10 upper***	0.58	0.86	1.69	1.01	0.23	0.38
**T10 middle***	0.55	0.83	1.61	0.94	0.26	0.46
**T10 lower***	0.60	0.88	1.52	0.88	0.30	0.53
**T11 upper***	0.61	0.88	1.45	0.84	0.32	0.57
**T11 middle***	0.61	0.88	1.33	0.78	0.38	0.67
**T11 lower***	0.66	0.93	1.25	0.76	0.45	0.80
**T12 upper***	0.67	0.95	1.23	0.75	0.50	0.89
**T12 middle***	0.71	0.99	1.19	0.73	0.60	1.04
**T12 lower***	0.74	1.03	1.20	0.74	0.66	1.14
**L1 upper***	0.77	1.06	1.20	0.73	0.71	1.20
**L1 middle***	0.81	1.12	1.28	0.78	0.76	1.27
**L1 lower***	0.84	1.16	1.37	0.83	0.79	1.32
**L2 upper***	0.85	1.17	1.43	0.88	0.80	1.34
**L2 middle***	0.88	1.23	1.52	0.96	0.83	1.36
**L2 lower***	0.92	1.30	1.57	1.04	0.83	1.36
**L3 upper***	0.93	1.33	1.60	1.10	0.84	1.36
**L3 middle***	0.95	1.38	1.63	1.21	0.84	1.35
**L3 lower***	0.99	1.43	1.69	1.33	0.82	1.28
**L4 upper***	0.97	1.40	1.77	1.41	0.80	1.17
**L4 middle***	0.96	1.38	1.92	1.50	0.77	1.04
**L4 lower***	0.94	1.29	2.06	1.56	0.75	0.92
**L5 upper***	0.89	1.21	2.14	1.57	0.72	0.82
**L5 middle***	0.86	1.23	2.19	1.53	0.70	0.76
**L5 lower***	0.95	1.33	2.17	1.45	0.67	0.71

* Single-slice measurement.

Supplementary Table 2. Summary† of Pearson correlation coefficients between thoracolumbar and whole-body compositions.

	Muscle		Visceral fat		Subcutaneous fat	
	**Female**	**Male**	**Female**	**Male**	**Female**	**Male**
**T1 upper***	0.66 [0.51, 0.77]	0.62 [0.46, 0.74]	0.26 [0.00, 0.48]	Not applicable‡	0.74 [0.65, 0.82]	0.80 [0.72, 0.87]
**T1 middle***	0.71 [0.58, 0.81]	0.64 [0.48, 0.76]	0.39 [0.21, 0.54]	0.25 [0.04, 0.45]	0.72 [0.64, 0.82]	0.83 [0.76, 0.89]
**T1 lower***	0.75 [0.65, 0.83]	0.66 [0.51, 0.77]	0.63 [0.50, 0.73]	0.64 [0.51, 0.74]	0.75 [0.66, 0.85]	0.85 [0.77, 0.91]
**T2 upper***	0.75 [0.64, 0.83]	0.69 [0.56, 0.79]	0.60 [0.49, 0.71]	0.69 [0.58, 0.78]	0.77 [0.68, 0.87]	0.85 [0.76, 0.91]
**T2 middle***	0.73 [0.60, 0.82]	0.69 [0.53, 0.80]	0.64 [0.54, 0.75]	0.73 [0.65, 0.80]	0.77 [0.69, 0.87]	0.85 [0.75, 0.92]
**T2 lower***	0.71 [0.57, 0.82]	0.68 [0.51, 0.80]	0.57 [0.48, 0.69]	0.63 [0.48, 0.75]	0.76 [0.68, 0.86]	0.86 [0.77, 0.92]
**T3 upper***	0.72 [0.59, 0.82]	0.71 [0.55, 0.82]	0.55 [0.43, 0.70]	0.60 [0.46, 0.71]	0.75 [0.67, 0.86]	0.86 [0.78, 0.92]
**T3 middle***	0.72 [0.63, 0.80]	0.78 [0.64, 0.86]	0.62 [0.45, 0.81]	0.67 [0.59, 0.75]	0.74 [0.66, 0.85]	0.87 [0.79, 0.92]
**T3 lower***	0.76 [0.67, 0.84]	0.79 [0.66, 0.88]	0.75 [0.63, 0.84]	0.72 [0.63, 0.81]	0.76 [0.69, 0.85]	0.86 [0.78, 0.92]
**T4 upper***	0.75 [0.65, 0.83]	0.78 [0.63, 0.88]	0.74 [0.62, 0.84]	0.75 [0.66, 0.84]	0.76 [0.69, 0.85]	0.85 [0.75, 0.92]
**T4 middle***	0.70 [0.58, 0.79]	0.79 [0.64, 0.89]	0.83 [0.76, 0.90]	0.76 [0.65, 0.84]	0.76 [0.69, 0.85]	0.85 [0.76, 0.92]
**T4 lower***	0.65 [0.52, 0.75]	0.76 [0.63, 0.86]	0.81 [0.73, 0.88]	0.77 [0.64, 0.86]	0.77 [0.70, 0.85]	0.86 [0.78, 0.93]
**T5 upper***	0.66 [0.54, 0.75]	0.73 [0.57, 0.84]	0.81 [0.72, 0.88]	0.78 [0.67, 0.87]	0.78 [0.71, 0.85]	0.87 [0.79, 0.93]
**T5 middle***	0.62 [0.51, 0.72]	0.70 [0.54, 0.81]	0.83 [0.76, 0.89]	0.81 [0.73, 0.88]	0.78 [0.72, 0.85]	0.87 [0.80, 0.93]
**T5 lower***	0.57 [0.42, 0.68]	0.67 [0.54, 0.78]	0.82 [0.76, 0.87]	0.77 [0.69, 0.85]	0.80 [0.73, 0.86]	0.89 [0.82, 0.93]
**T6 upper***	0.55 [0.40, 0.68]	0.66 [0.52, 0.77]	0.77 [0.70, 0.85]	0.78 [0.71, 0.86]	0.80 [0.73, 0.87]	0.89 [0.83, 0.93]
**T6 middle***	0.60 [0.45, 0.74]	0.67 [0.53, 0.78]	0.80 [0.73, 0.85]	0.79 [0.70, 0.87]	0.81 [0.74, 0.87]	0.89 [0.84, 0.93]
**T6 lower***	0.59 [0.44, 0.73]	0.69 [0.55, 0.80]	0.76 [0.66, 0.85]	0.70 [0.58, 0.81]	0.81 [0.74, 0.87]	0.90 [0.84, 0.94]
**T7 upper***	0.59 [0.43, 0.74]	0.70 [0.56, 0.80]	0.74 [0.63, 0.84]	0.70 [0.58, 0.79]	0.79 [0.72, 0.86]	0.90 [0.85, 0.94]
**T7 middle***	0.60 [0.43, 0.78]	0.70 [0.55, 0.81]	0.77 [0.67, 0.86]	0.78 [0.69, 0.85]	0.80 [0.73, 0.86]	0.91 [0.86, 0.95]
**T7 lower***	0.66 [0.48, 0.82]	0.74 [0.59, 0.84]	0.72 [0.58, 0.81]	0.79 [0.68, 0.86]	0.79 [0.72, 0.85]	0.92 [0.88, 0.95]
**T8 upper***	0.62 [0.44, 0.81]	0.72 [0.57, 0.83]	0.70 [0.63, 0.79]	0.79 [0.69, 0.85]	0.79 [0.71, 0.85]	0.92 [0.88, 0.95]
**T8 middle***	0.62 [0.47, 0.78]	0.69 [0.53, 0.80]	0.73 [0.69, 0.82]	0.82 [0.76, 0.87]	0.78 [0.70, 0.84]	0.92 [0.88, 0.95]
**T8 lower***	0.64 [0.49, 0.81]	0.70 [0.55, 0.82]	0.77 [0.70, 0.84]	0.81 [0.73, 0.87]	0.77 [0.69, 0.84]	0.92 [0.88, 0.95]
**T9 upper***	0.67 [0.54, 0.80]	0.72 [0.58, 0.82]	0.77 [0.67, 0.85]	0.80 [0.72, 0.87]	0.77 [0.68, 0.84]	0.92 [0.88, 0.95]
**T9 middle***	0.64 [0.49, 0.79]	0.71 [0.57, 0.81]	0.76 [0.67, 0.84]	0.77 [0.67, 0.85]	0.79 [0.70, 0.85]	0.90 [0.86, 0.94]

**T9 lower***	0.78 [0.69, 0.85]	0.72 [0.58, 0.82]	0.81 [0.74, 0.86]	0.77 [0.67, 0.86]	0.78 [0.70, 0.85]	0.91 [0.87, 0.94]
**T10 upper***	0.75 [0.65, 0.83]	0.72 [0.58, 0.83]	0.81 [0.74, 0.87]	0.80 [0.70, 0.87]	0.78 [0.69, 0.84]	0.91 [0.87, 0.94]
**T10 middle***	0.71 [0.56, 0.81]	0.74 [0.62, 0.83]	0.83 [0.77, 0.87]	0.82 [0.76, 0.89]	0.77 [0.67, 0.84]	0.91 [0.87, 0.94]
**T10 lower***	0.77 [0.66, 0.85]	0.78 [0.68, 0.85]	0.83 [0.79, 0.87]	0.83 [0.77, 0.89]	0.75 [0.65, 0.83]	0.91 [0.87, 0.94]
**T11 upper***	0.77 [0.66, 0.85]	0.79 [0.71, 0.86]	0.86 [0.82, 0.90]	0.83 [0.76, 0.89]	0.76 [0.65, 0.83]	0.91 [0.87, 0.94]
**T11 middle***	0.79 [0.69, 0.86]	0.80 [0.71, 0.87]	0.88 [0.84, 0.92]	0.81 [0.71, 0.89]	0.75 [0.65, 0.83]	0.91 [0.87, 0.94]
**T11 lower***	0.80 [0.70, 0.87]	0.80 [0.70, 0.88]	0.91 [0.88, 0.95]	0.85 [0.78, 0.91]	0.76 [0.67, 0.84]	0.92 [0.89, 0.95]
**T12 upper***	0.79 [0.69, 0.86]	0.79 [0.69, 0.87]	0.92 [0.89, 0.95]	0.87 [0.80, 0.92]	0.77 [0.68, 0.84]	0.93 [0.89, 0.95]
**T12 middle***	0.80 [0.71, 0.86]	0.78 [0.68, 0.85]	0.92 [0.89, 0.95]	0.90 [0.85, 0.94]	0.82 [0.75, 0.87]	0.93 [0.90, 0.95]
**T12 lower***	0.79 [0.71, 0.86]	0.78 [0.66, 0.86]	0.93 [0.90, 0.95]	0.91 [0.85, 0.95]	0.84 [0.78, 0.89]	0.93 [0.90, 0.96]
**Multi-slice T12-L1 averaging**	0.82 [0.75, 0.88]	0.81 [0.68, 0.89]	0.95 [0.93, 0.97]	0.95 [0.92, 0.97]	0.85 [0.79, 0.90]	0.93 [0.90, 0.96]
**L1 upper***	0.81 [0.73, 0.87]	0.83 [0.75, 0.89]	0.94 [0.92, 0.96]	0.95 [0.92, 0.96]	0.86 [0.80, 0.90]	0.93 [0.90, 0.96]
**L1 middle***	0.83 [0.77, 0.89]	0.82 [0.72, 0.88]	0.95 [0.94, 0.97]	0.96 [0.94, 0.97]	0.86 [0.80, 0.90]	0.92 [0.89, 0.96]
**L1 lower***	0.83 [0.76, 0.88]	0.82 [0.71, 0.89]	0.97 [0.95, 0.98]	0.97 [0.96, 0.98]	0.86 [0.80, 0.90]	0.92 [0.88, 0.95]
**L2 upper***	0.83 [0.76, 0.89]	0.81 [0.67, 0.89]	0.97 [0.95, 0.98]	0.97 [0.96, 0.98]	0.87 [0.81, 0.91]	0.92 [0.88, 0.95]
**L2 middle***	0.83 [0.76, 0.89]	0.81 [0.68, 0.90]	0.97 [0.95, 0.98]	0.98 [0.96, 0.99]	0.88 [0.84, 0.92]	0.91 [0.87, 0.95]
**L2 lower***	0.85 [0.79, 0.90]	0.83 [0.72, 0.90]	0.97 [0.96, 0.98]	0.97 [0.96, 0.98]	0.88 [0.83, 0.91]	0.90 [0.85, 0.94]
**L3 upper***	0.86 [0.80, 0.91]	0.84 [0.73, 0.90]	0.97 [0.96, 0.98]	0.97 [0.96, 0.98]	0.86 [0.81, 0.91]	0.90 [0.85, 0.93]
**L3 middle***	0.85 [0.78, 0.90]	0.86 [0.78, 0.92]	0.97 [0.95, 0.98]	0.97 [0.96, 0.98]	0.84 [0.78, 0.89]	0.89 [0.84, 0.93]
**L3 lower***	0.85 [0.79, 0.90]	0.86 [0.79, 0.91]	0.96 [0.95, 0.97]	0.96 [0.94, 0.98]	0.84 [0.77, 0.88]	0.89 [0.84, 0.94]
**L4 upper***	0.85 [0.78, 0.90]	0.87 [0.81, 0.92]	0.96 [0.94, 0.97]	0.94 [0.92, 0.97]	0.84 [0.77, 0.89]	0.90 [0.86, 0.94]
**L4 middle***	0.81 [0.72, 0.88]	0.87 [0.81, 0.91]	0.95 [0.92, 0.97]	0.94 [0.90, 0.96]	0.85 [0.78, 0.90]	0.92 [0.87, 0.95]
**L4 lower***	0.80 [0.72, 0.86]	0.85 [0.79, 0.90]	0.96 [0.93, 0.97]	0.93 [0.90, 0.96]	0.87 [0.81, 0.91]	0.93 [0.90, 0.96]
**L5 upper***	0.80 [0.71, 0.85]	0.86 [0.80, 0.90]	0.95 [0.93, 0.97]	0.91 [0.87, 0.95]	0.89 [0.85, 0.93]	0.93 [0.90, 0.96]
**L5 middle***	0.77 [0.68, 0.84]	0.82 [0.73, 0.87]	0.96 [0.94, 0.97]	0.91 [0.87, 0.95]	0.91 [0.87, 0.94]	0.94 [0.90, 0.96]
**L5 lower***	0.68 [0.56, 0.77]	0.73 [0.61, 0.81]	0.95 [0.94, 0.97]	0.90 [0.85, 0.94]	0.91 [0.87, 0.94]	0.93 [0.90, 0.96]

**Multi-slice waist averaging**	0.88 [0.82, 0.92]	0.91 [0.87, 0.93]	0.98 [0.97, 0.99]	0.97 [0.95, 0.98]	0.91 [0.88, 0.94]	0.91 [0.85, 0.94]

Data in parentheses indicate 95% confidence intervals.

† Pearson correlation coefficients and 95% confidence intervals based on 1,000 bootstrap resamples.

* Single-slice measurement.

‡ The coefficient could not be assessed, as the visceral fat amount at the single-slice T1 upper endplate level was zero in most male patients.

Supplementary Table 3. Correlation coefficients between thoracolumbar and whole-body compositions.

	Correlation coefficients [95% CI]
T1 upper*	0.50 [0.30, 0.52]
T1 middle*	0.59 [0.30, 0.87]
T1 lower*	0.71 [0.30, 0.89]
T2 upper*	0.72 [0.30, 0.89]
T2 middle*	0.73 [0.34, 0.89]
T2 lower*	0.70 [0.30, 0.88]
T3 upper*	0.70 [0.30, 0.88]
T3 middle*	0.73 [0.37, 0.90]
T3 lower*	0.77 [0.41, 0.90]
T4 upper*	0.77 [0.42, 0.90]
T4 middle*	0.78 [0.43, 0.90]
T4 lower*	0.77 [0.42, 0.90]
T5 upper*	0.77 [0.43, 0.90]
T5 middle*	0.77 [0.43, 0.90]
T5 lower*	0.75 [0.39, 0.90]
T6 upper*	0.74 [0.37, 0.89]
T6 middle*	0.76 [0.42, 0.90]
T6 lower*	0.74 [0.38, 0.89]
T7 upper*	0.74 [0.37, 0.89]
T7 middle*	0.76 [0.45, 0.90]
T7 lower*	0.77 [0.48, 0.91]
T8 upper*	0.76 [0.43, 0.90]
T8 middle*	0.76 [0.45, 0.90]
T8 lower*	0.77 [0.51, 0.91]
T9 upper*	0.78 [0.56, 0.91]
T9 middle*	0.76 [0.47, 0.90]
T9 lower*	0.80 [0.69, 0.92]
T10 upper*	0.80 [0.69, 0.92]
T10 middle*	0.80 [0.69, 0.92]
T10 lower*	0.81 [0.71, 0.92]
T11 upper*	0.82 [0.71, 0.92]
T11 middle*	0.83 [0.72, 0.92]
T11 lower*	0.84 [0.73, 0.92]
T12 upper*	0.85 [0.73, 0.92]
T12 middle*	0.86 [0.73, 0.92]
T12 lower*	0.86 [0.74, 0.92]
Multi-slice T12-L1 averaging	0.89 [0.77, 0.94]
L1 upper*	0.89 [0.86, 0.91]
L1 middle*	0.89 [0.87, 0.91]
L1 lower*	0.89 [0.87, 0.92]
L2 upper*	0.89 [0.87, 0.92]
L2 middle*	0.90 [0.88, 0.92]
L2 lower*	0.90 [0.88, 0.93]
L3 upper*	0.90 [0.88, 0.93]
L3 middle*	0.90 [0.87, 0.92]
L3 lower*	0.90 [0.87, 0.92]
L4 upper*	0.89 [0.86, 0.91]
L4 middle*	0.89 [0.85, 0.90]

**L4 lower***	0.89 [0.86, 0.91]
**L5 upper***	0.89 [0.86, 0.91]
**L5 middle***	0.88 [0.84, 0.90]
**L5 lower***	0.85 [0.80, 0.86]
**Multi-slice waist averaging**	0.92 [0.92, 0.95]

* Single-slice measurement.

Supplementary Table 4. Coefficients of final prediction model for whole body muscle.

	MSA	L1 upper	L2 lower	L3 upper	MSL	T12 lower
Intercept	-22277987.4	-22934664.3	-25043010.5	-22548304.2	-19010898.5	-20836957.2
Muscle composition volume (liter)	1296.3	1347.7	1146.4	1069.6	1168.0	1096.3
Female	11770916.5	11333776.1	15233814.3	12260153.5	9307774.9	10441172.4
Age (year)	0	-48396.6	-33005.6	-32027.8	-57333.3	-56231.9
Height (cm)	149019.7	194322.5	193278.6	173647.7	180616.8	198063.7
Weight (kg)	41155.4	34922.8	44123.1	65733.9	46298.3	44958.8
Female * CT slice muscle volume [a]	-232.5	-261.4	-232.8	0	0	0
Female * Age [a]	0	32673.9	24658.0	25693.7	41456.2	37476.2
Female * Height [a]	-75643.2	-76562.4	-109130.7	-89066.5	-82448.5	-89195.5
Female * Weight [a]	46388.7	0	43480.5	0	0	0

Note: upper, upper endplate; lower, lower endplate; MSA, multi-slice 3D analysis of abdomen; MSL, multi-slice 3D analysis of lower thorax, T12-L1 areas.

[a] interaction term which consider relationship between body composition according to sex.

Supplementary Table 5. Coefficients of final prediction model for whole body VF.

	MSA	L1 upper	L2 lower	L3 upper	MSL	T12 lower
Intercept	-2184375.4	-4664612.9	-4081456.2	-3547787.4	-4164625.0	-4978681.8
VF composition volume (liter)	254.9	231.3	242.2	234.0	238.4	203.8
Female	1086554.7	530949.8	3353041.7	2666579.4	416382.3	352822.1
Age (year)	0	10129.2	3942.7	6500.1	8667.8	15800.1
Height (cm)	8222.2	19890.4	24252.2	18075.4	18956.3	19496.5
Weight (kg)	18041.8	21325.5	0	6765.5	17162.8	28308.5
Female * CT slice volume of VF [a]	23.9	-17.3	0	0	0	0
Female * Age [a]	0	0	0	0	0	0
Female * Height [a]	0	0	-18914.4	-14920.2	0	0
Female * Weight [a]	-19954.9	0	0	0	0	0

Note: VF, visceral fat; upper, upper endplate; lower, lower endplate; MSA, multi-slice 3D analysis of abdomen; MSL, multi-slice 3D analysis of lower thorax, T12-L1 areas.

[a] interaction term which consider relationship between body composition according to sex.

Supplementary Table 6. Coefficients of final prediction model for whole body SF.

	MSA	L1 upper	L2 lower	L3 upper	MSL	T12 lower
Intercept	-7278441.5	-2219975.8	-6121745.0	311980.7	-2891435.7	-2445560.0
SF composition volume (liter)	687.3	1114.3	755.3	700.6	1100.1	1130.5
Female	2595053.8	887012.9	567609.0	-339200.6	1168692.2	78949.3
Age (year)	49325.3	11215.3	31433.3	24911.1	12541.5	10225.8
Height (cm)	0	0	0	-41141.5	0	0
Weight (kg)	126713.3	101402.9	144494.9	160627.7	106262.5	102454.6
Female * CT slice volume of SF [a]	-164.7	-543.8	-314.5	-322.2	-535.4	-590.2
Female * Age [a]	-68979.4	-66749.1	-44614.6	-41361.0	-67485.3	-60481.8
Female * Height [a]	0	0	0	0	0	0
Female * Weight [a]	130330.8	173696.5	160634.6	178742.9	172767.1	192202.9

Note: SF, subcutaneous fat; upper, upper endplate; lower, lower endplate; MSA, multi-slice 3D analysis of abdomen; MSL, multi-slice 3D analysis of lower thorax, T12-L1 areas.

[a] interaction term which consider relationship between body composition according to sex.

Supplementary Figure 1

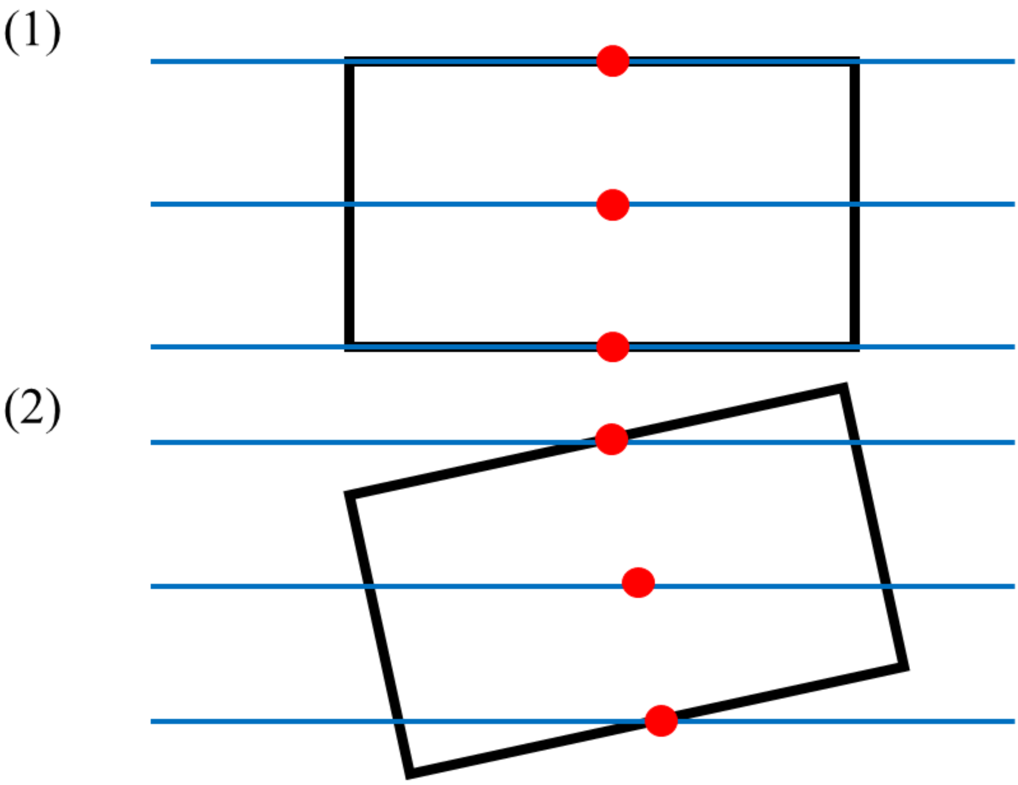

Supplementary Figure 1. A diagram showing how the vertebral levels were set at the upper, lower endplate, and mid-vertebra between the T1 and L5 vertebrae.

(1) If the vertebra was not tilted, the upper and lower endplates were set as the upper/lower regions of the vertebral body where a vertebral body and intervertebral disc interfaced. The mid-vertebra was defined by the midline of the upper and lower endplates. (2) If the vertebral body was tilted, the upper and lower endplates were measured based on the slice containing the center of the anteroposterior plane. The mid-vertebra was defined by the midline of the upper and lower endplates.

Supplementary Figure 2 (a)

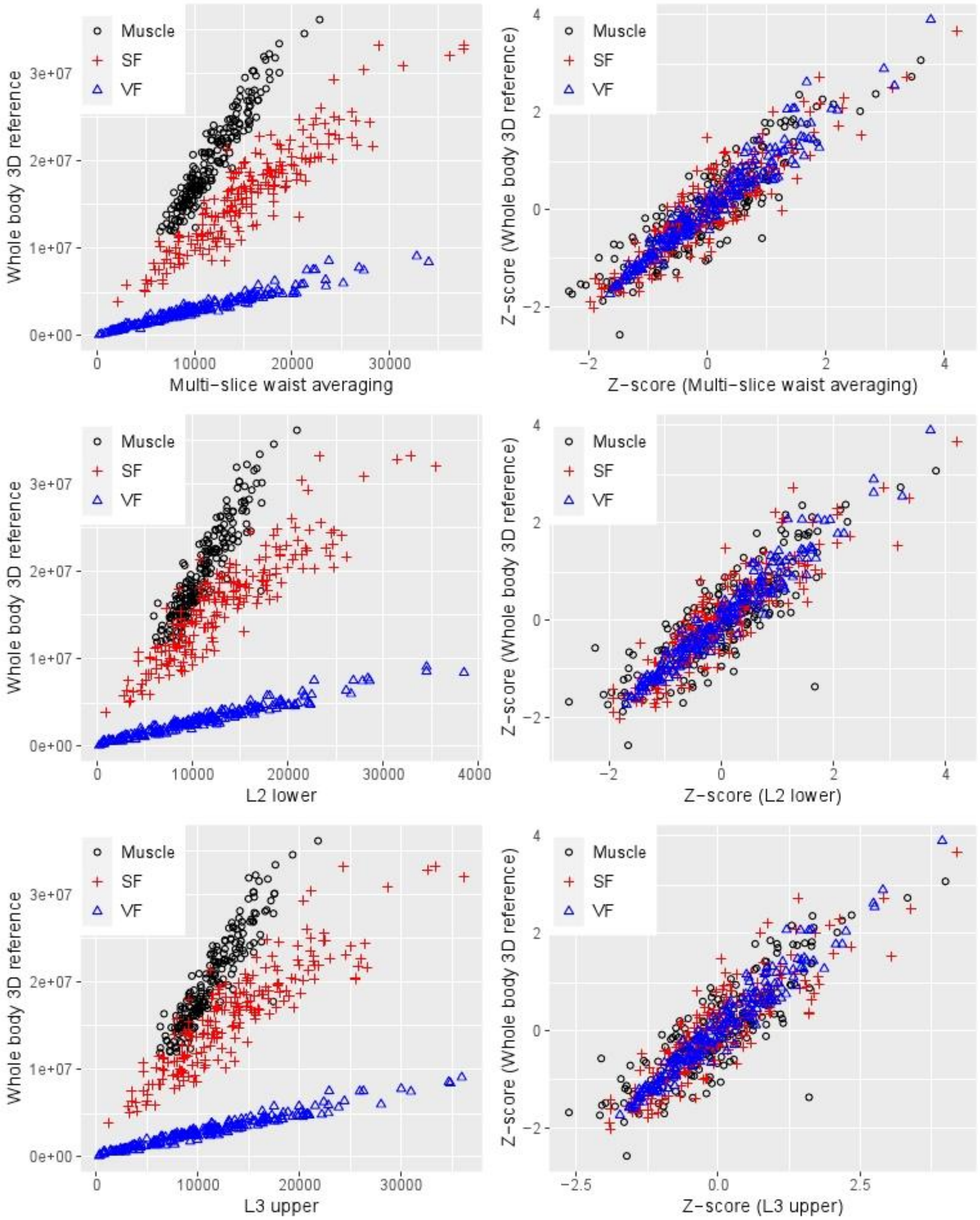

Supplementary Figure 2. Scatter plots of whole-body composition versus CT slice-based composition (left: measurements in original scale, right: standardized score).

Supplementary Figure 2 (b)

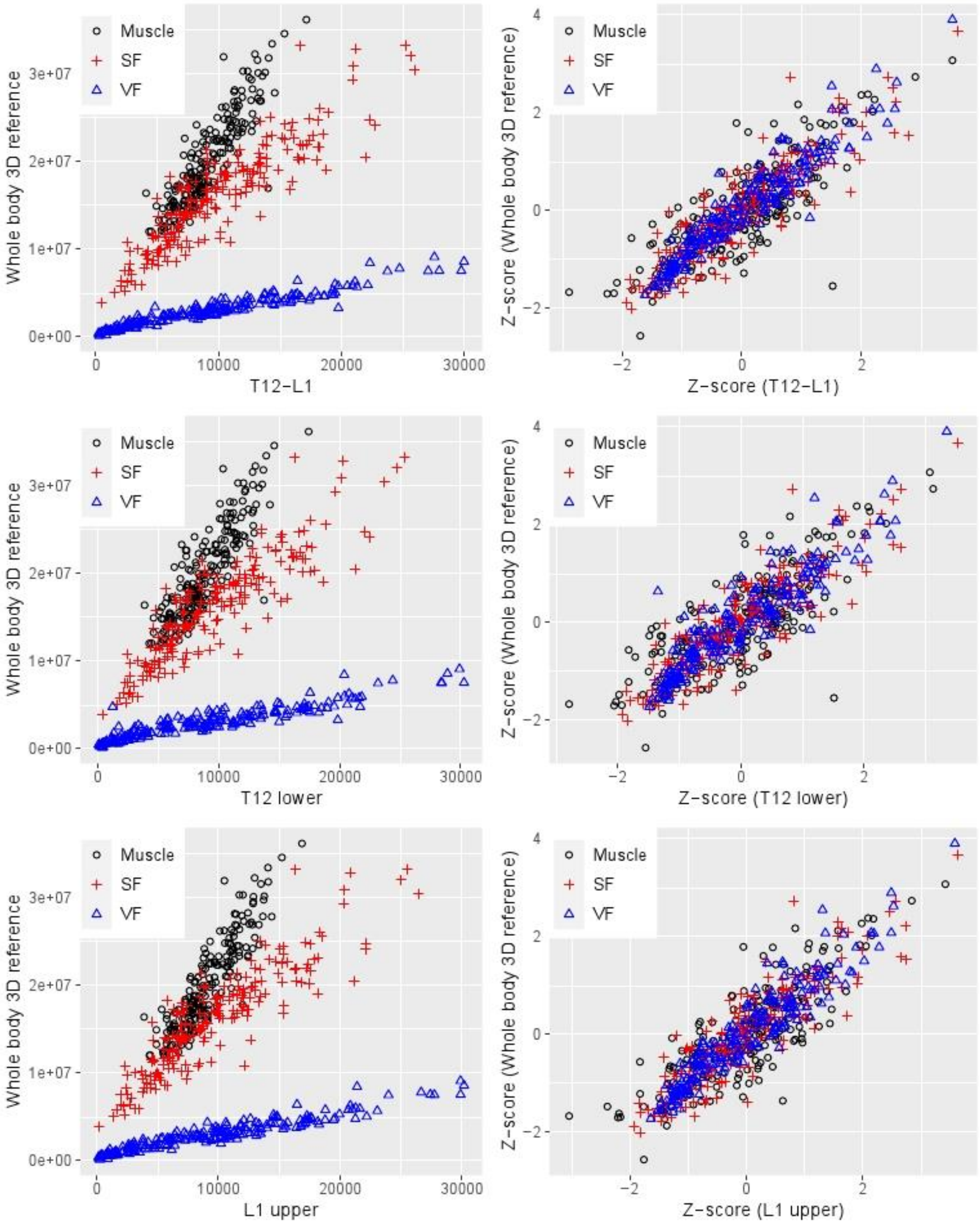

Supplementary Figure 2. Scatter plots of whole-body composition versus CT slice-based composition (left: measurements in original scale, right: standardized score).

Supplementary Figure 3 (a)

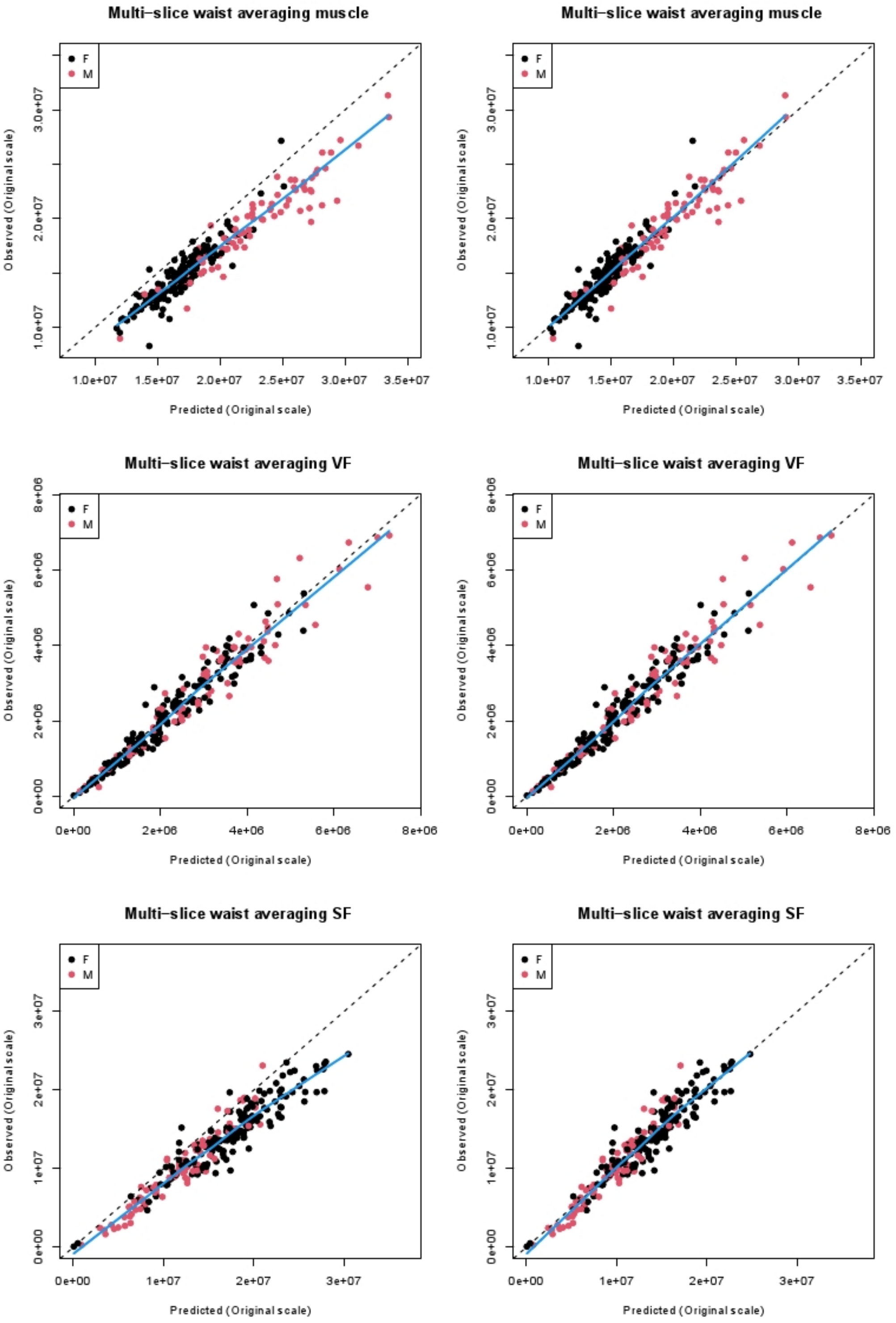

Supplementary Figure 3. Observed versus predicted whole-body muscle, VF, and SF before (left) and after (right) updating prediction models in the validation dataset. The dashed black line indicates unity (y=x) and the solid blue line represents a locally weighted scatterplot smoothing curve fitted to the data points.

Supplementary Figure 3 (b)

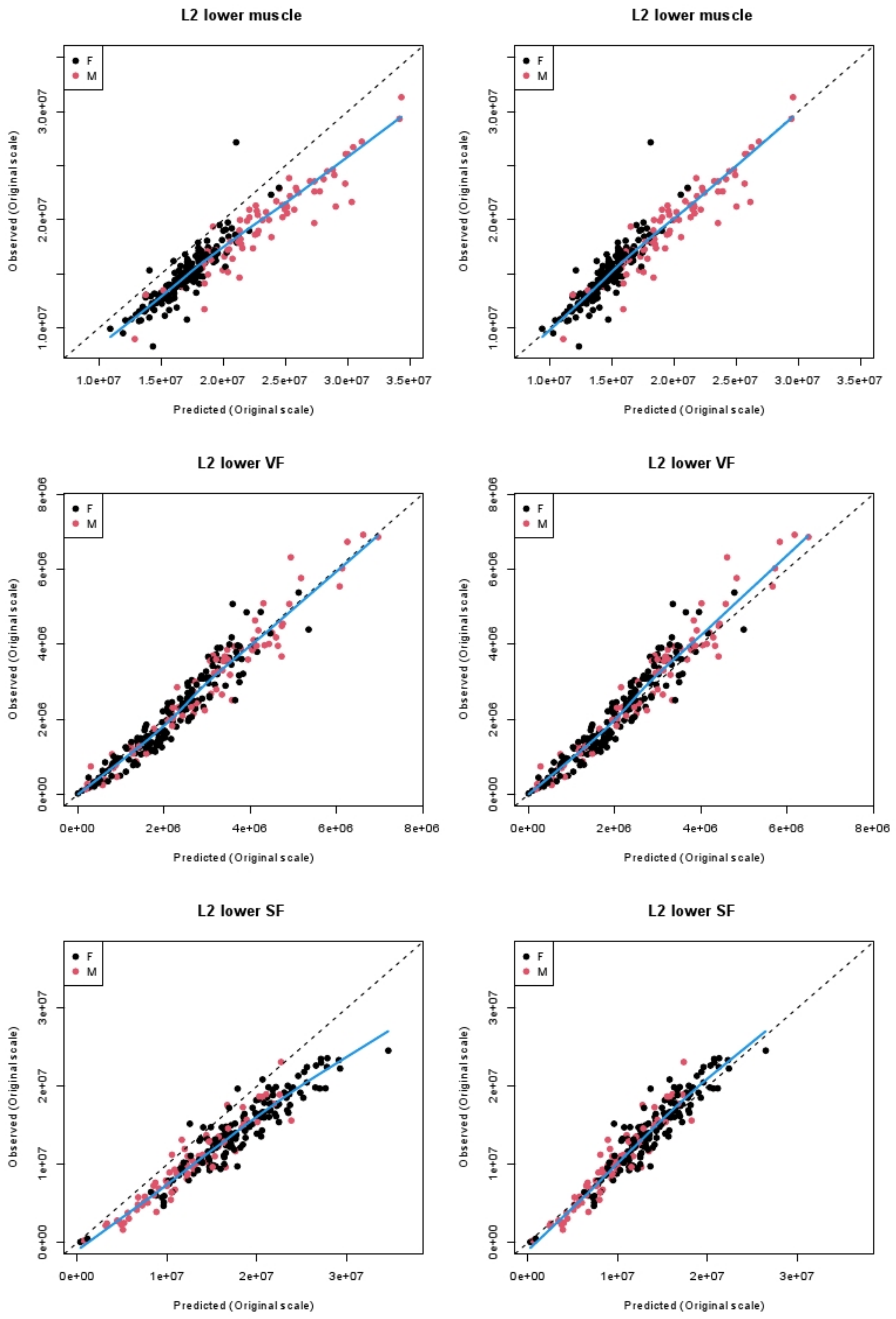

Supplementary Figure 3. Observed versus predicted whole-body muscle, VF, and SF before (left) and after (right) updating prediction models in the validation dataset. The dashed black line indicates unity (y=x) and the solid blue line represents a locally weighted scatterplot smoothing curve fitted to the data points.

Supplementary Figure 3 (c)

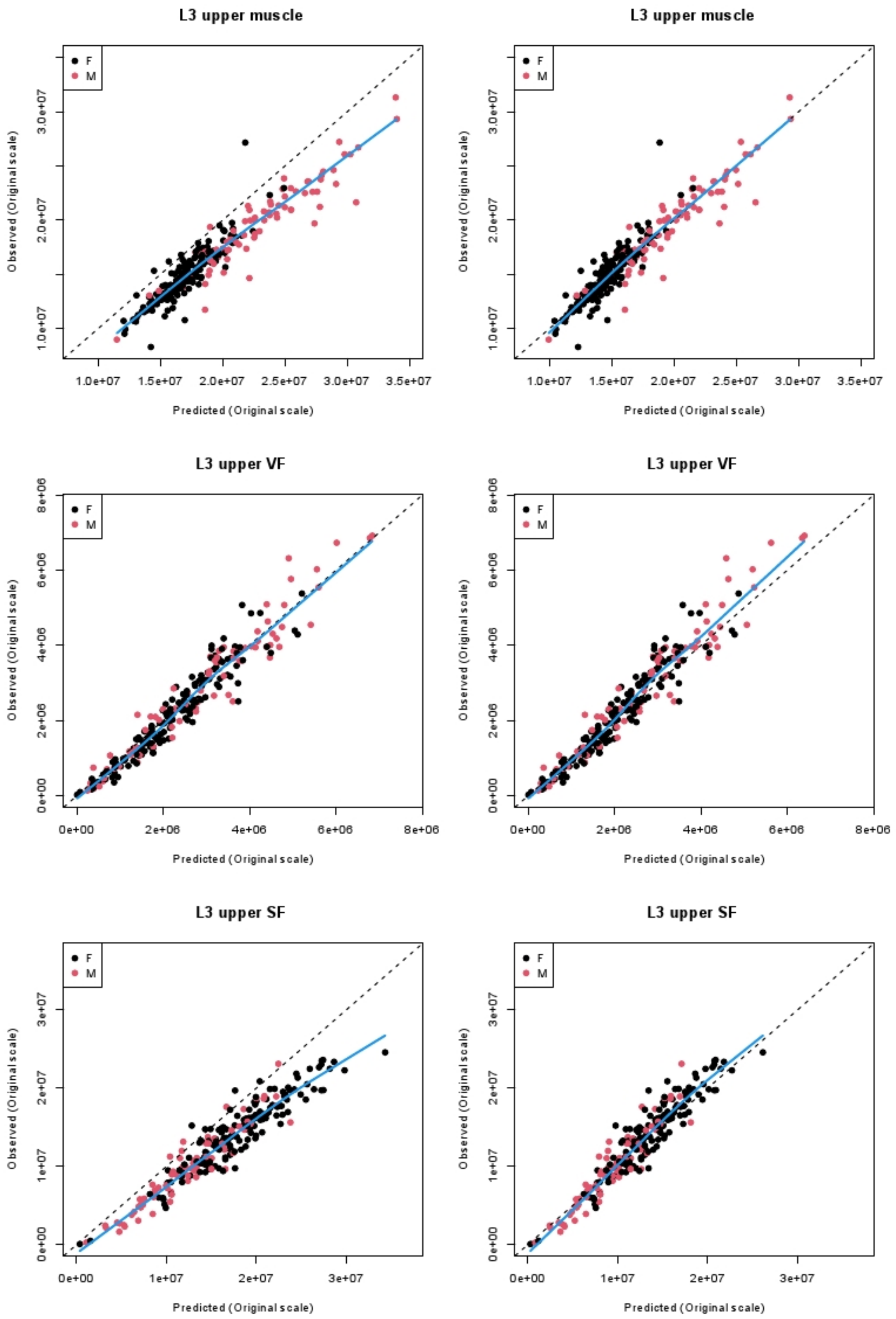

Supplementary Figure 3. Observed versus predicted whole-body muscle, VF, and SF before (left) and after (right) updating prediction models in the validation dataset. The dashed black line indicates unity (y=x) and the solid blue line represents a locally weighted scatterplot smoothing curve fitted to the data points.

Supplementary Figure 3 (d)

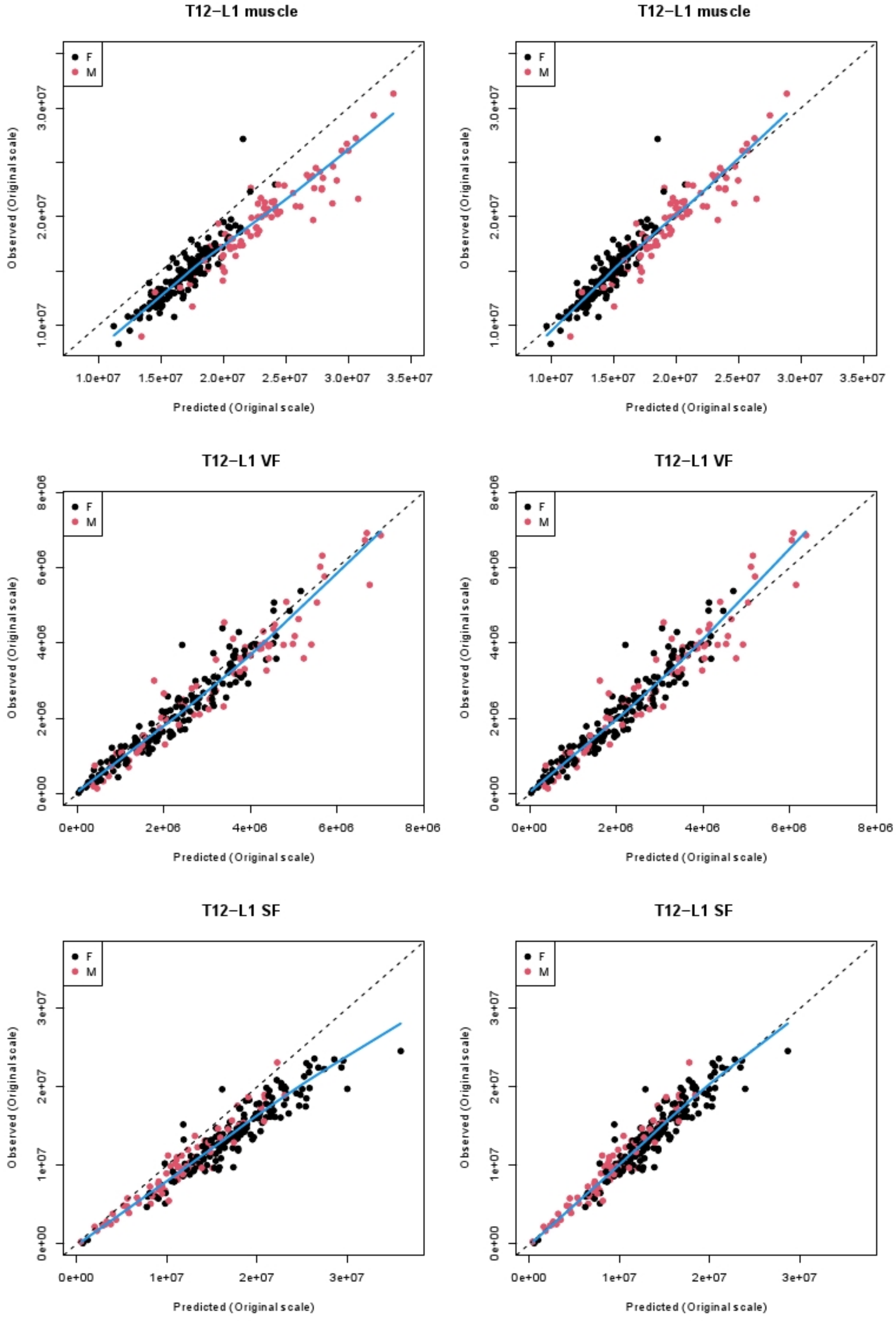

Supplementary Figure 3. Observed versus predicted whole-body muscle, VF, and SF before (left) and after (right) updating prediction models in the validation dataset. The dashed black line indicates unity (y=x) and the solid blue line represents a locally weighted scatterplot smoothing curve fitted to the data points.

Supplementary Figure 3 (e)

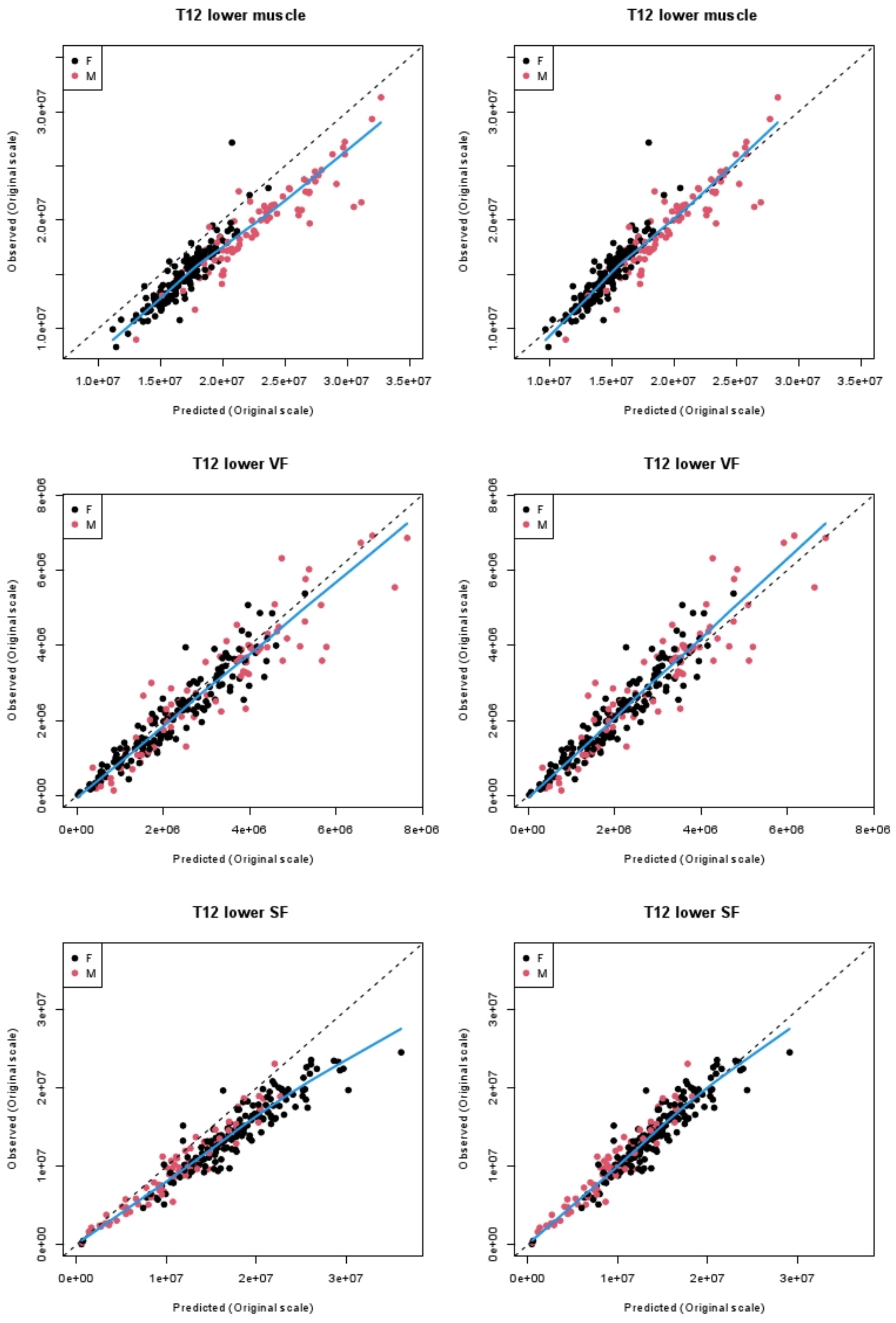

Supplementary Figure 3. Observed versus predicted whole-body muscle, VF, and SF before (left) and after (right) updating prediction models in the validation dataset. The dashed black line indicates unity (y=x) and the solid blue line represents a locally weighted scatterplot smoothing curve fitted to the data points.

Supplementary Figure 3 (f)

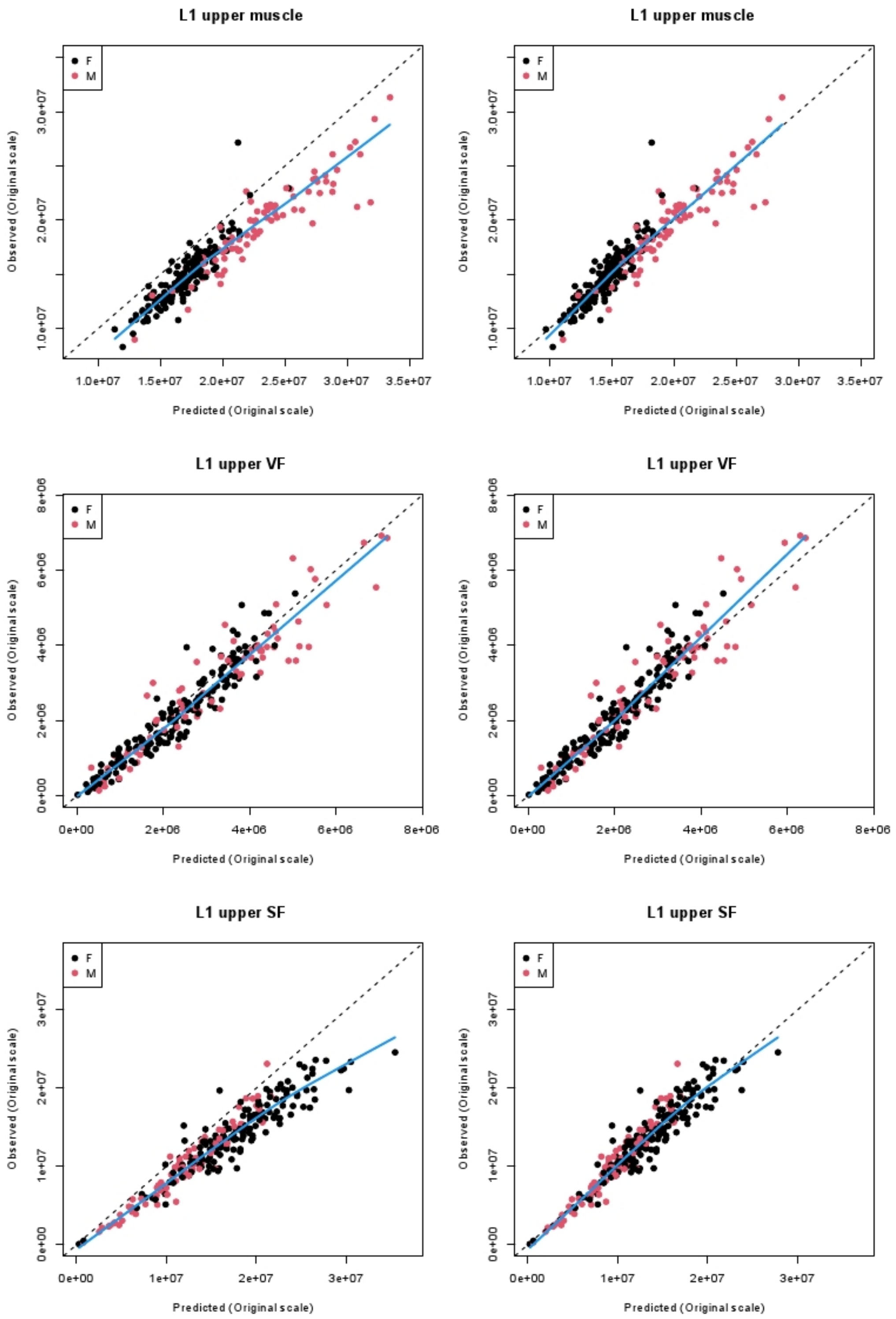

Supplementary Figure 3. Observed versus predicted whole-body muscle, VF, and SF before (left) and after (right) updating prediction models in the validation dataset. The dashed black line indicates unity (y=x) and the solid blue line represents a locally weighted scatterplot smoothing curve fitted to the data points.

Supplementary Figure 4 (a)

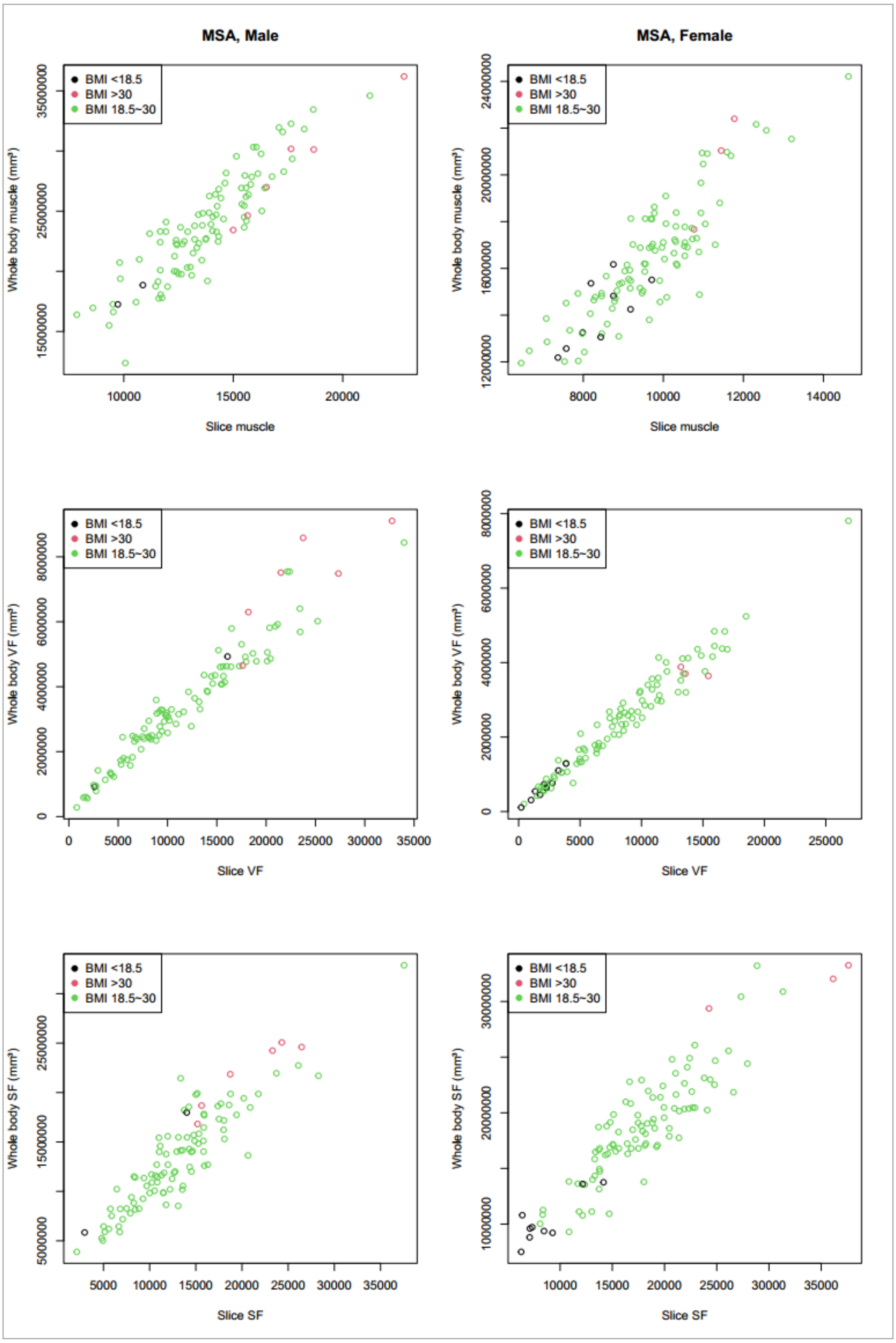

Supplementary Figure 4. Scatter plots of body composition in whole-body versus CT slice by BMI classification.

Supplementary Figure 4 (b)

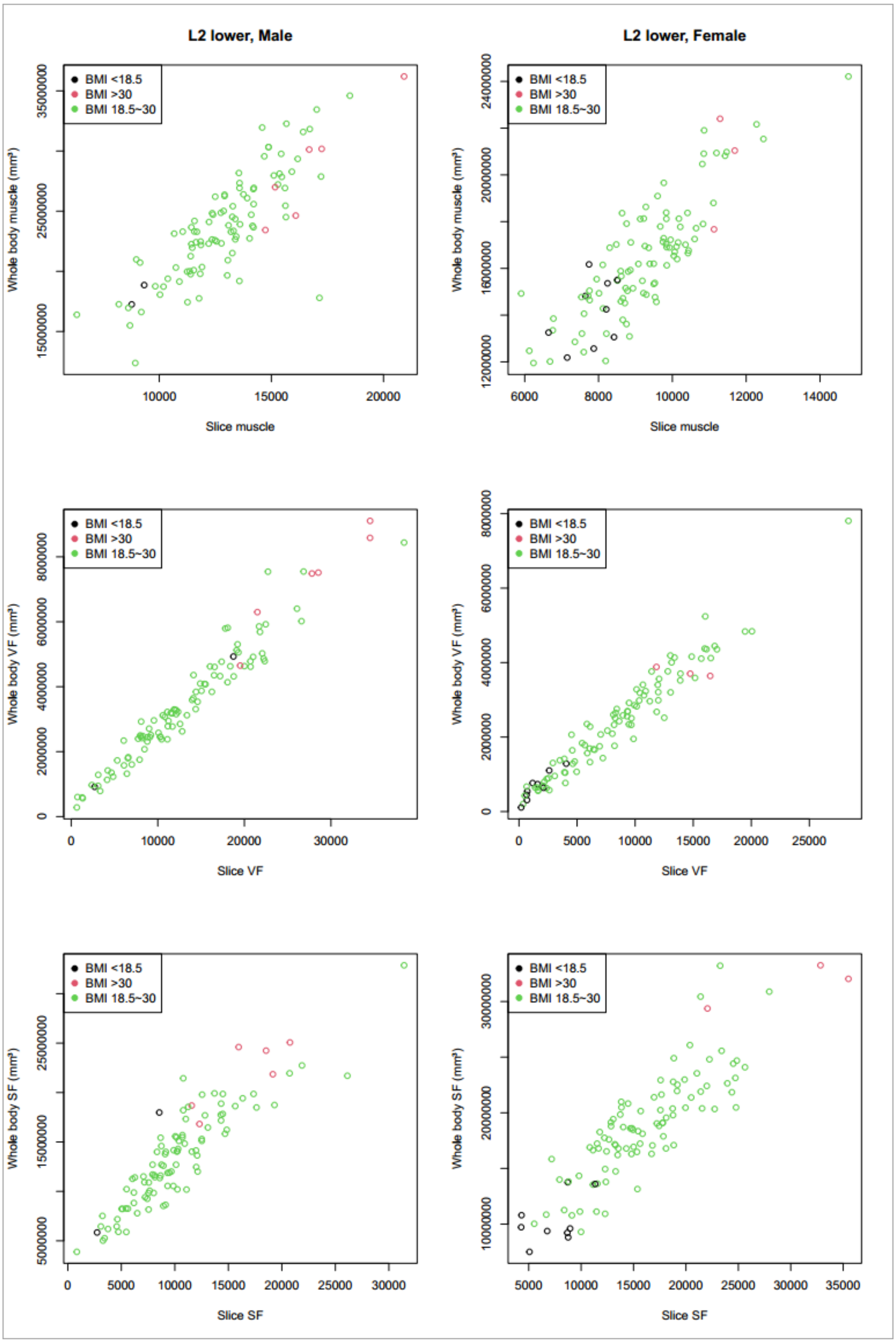

Supplementary Figure 4. Scatter plots of body composition in whole-body versus CT slice by BMI classification.

Supplementary Figure 4 (c)

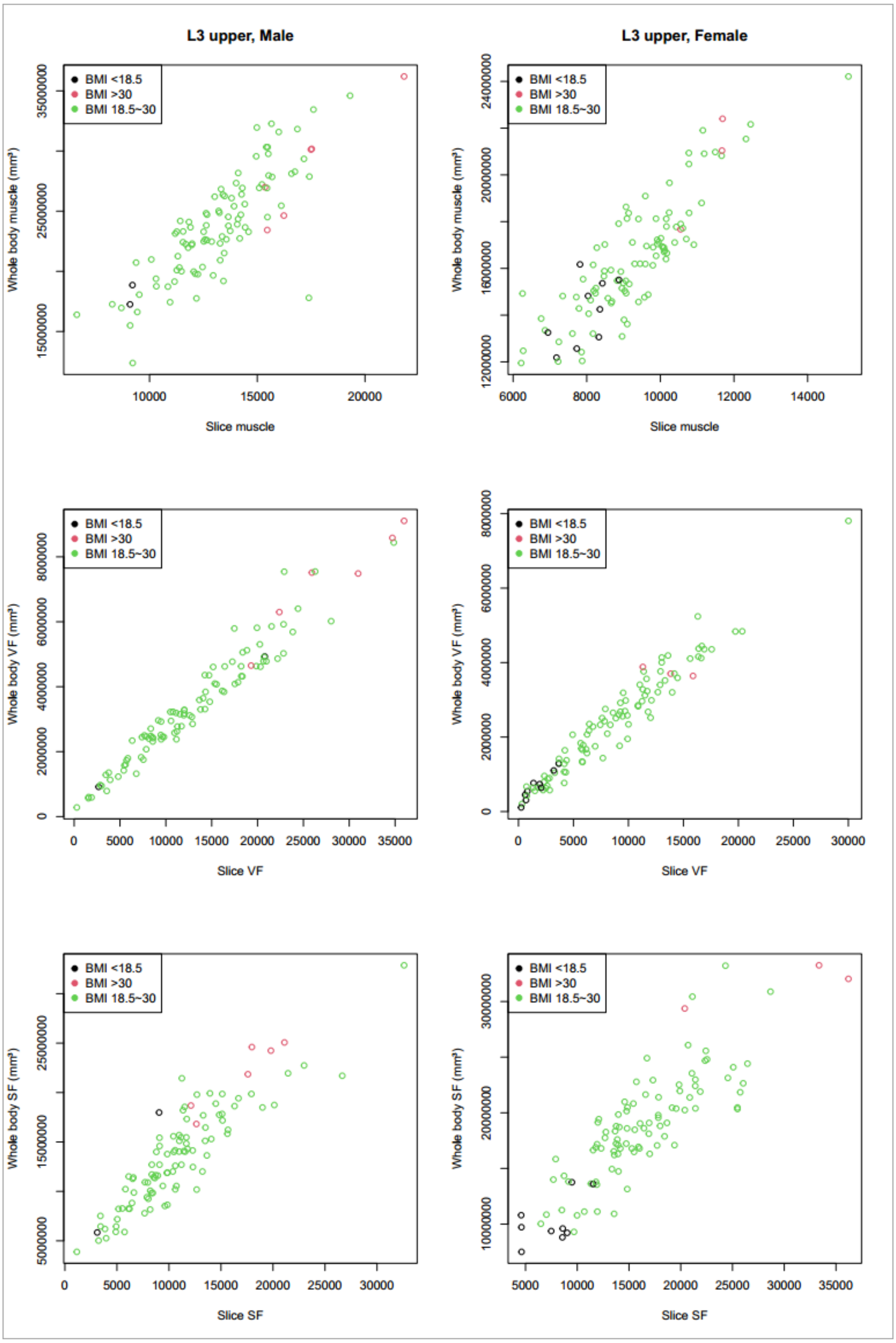

Supplementary Figure 4. Scatter plots of body composition in whole-body versus CT slice by BMI classification.

Supplementary Figure 4 (d)

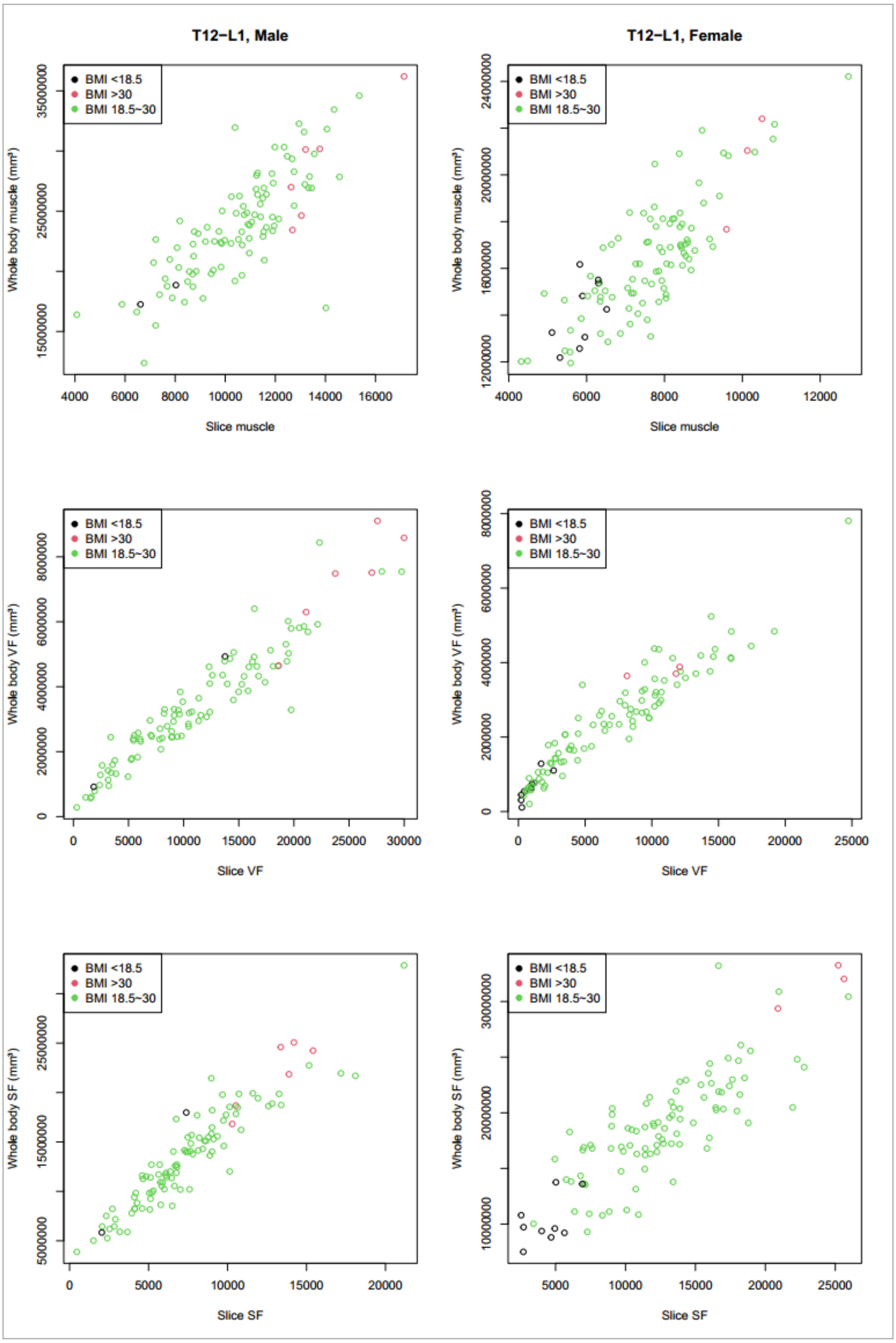

Supplementary Figure 4. Scatter plots of body composition in whole-body versus CT slice by BMI classification.

Supplementary Figure 4 (e)

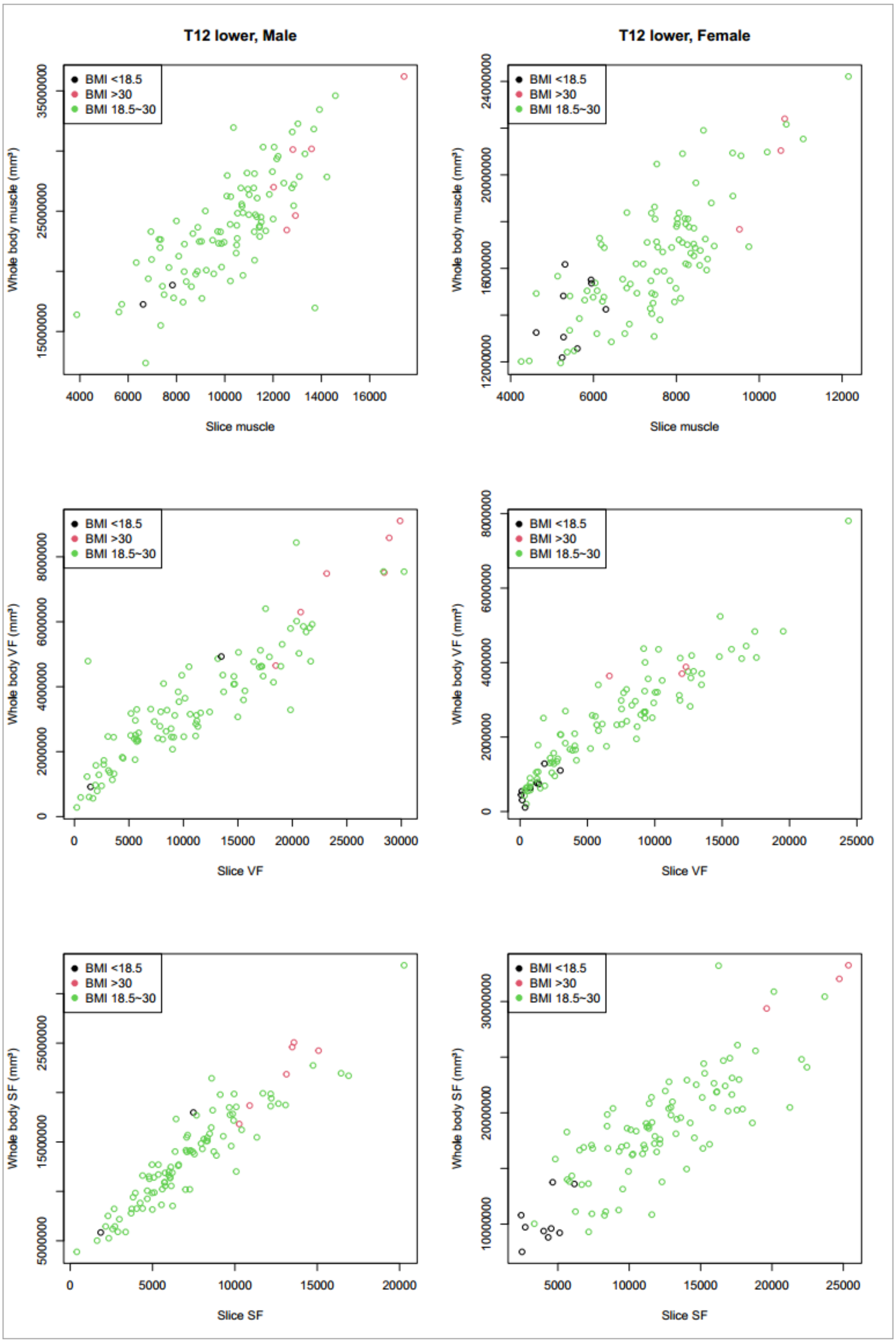

Supplementary Figure 4. Scatter plots of body composition in whole-body versus CT slice by BMI classification.

Supplementary Figure 4 (f)

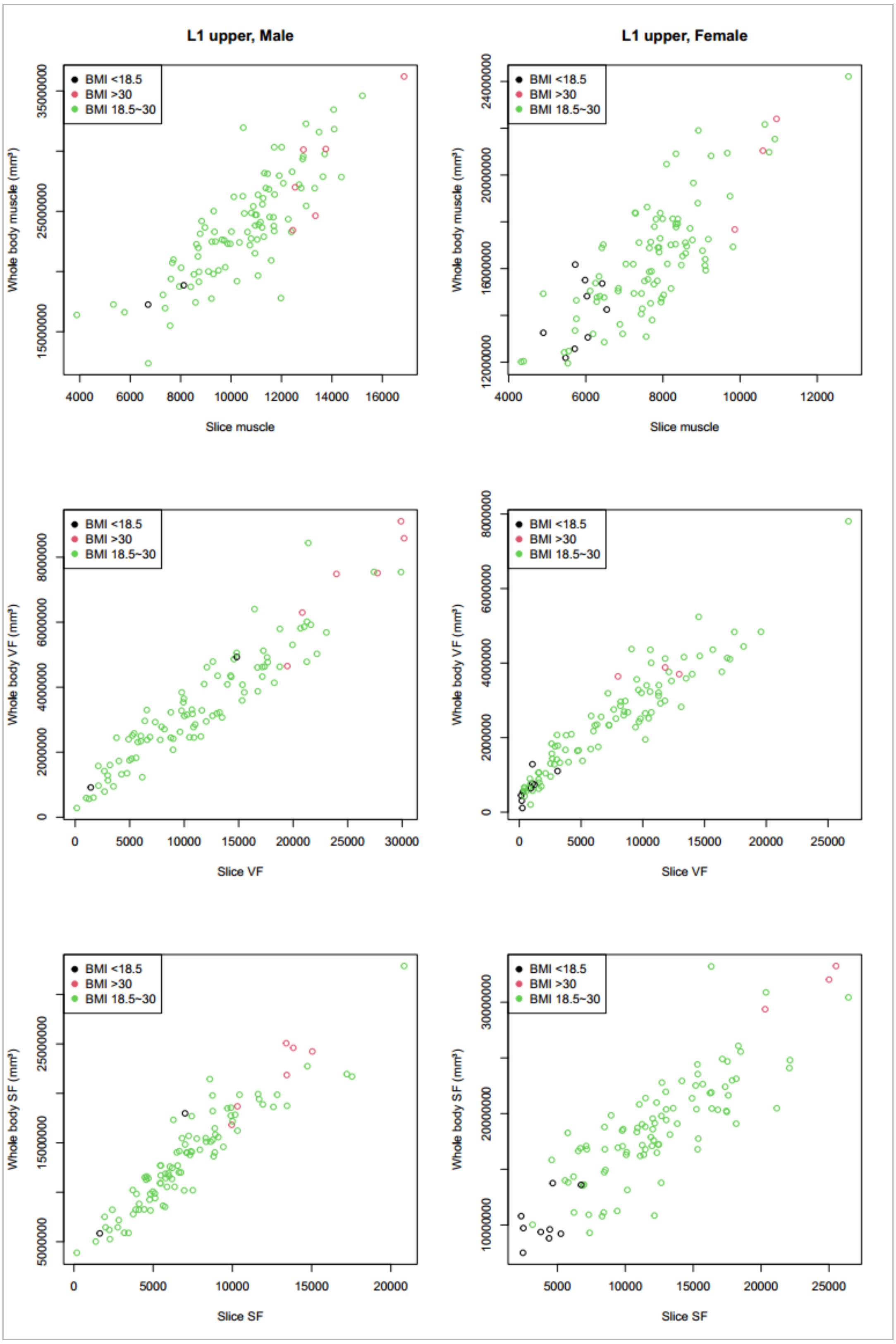

Supplementary Figure 4. Scatter plots of body composition in whole-body versus CT slice by BMI classification.
